# Pharmacokinetic modelling during long-term anesthesia: minimizing the gap^☆^

**DOI:** 10.1016/j.jare.2025.06.047

**Published:** 2025-06-24

**Authors:** Amani R. Ynineb, Erhan Yumuk, Dana Copot, Ghada Ben Othman, Hamed Farbakhsh, Isabela Birs, Robin De Keyser, Samir Ladaci, Cristina Muresan, Martine Neckebroek, Clara M. Ionescu

**Affiliations:** a Ghent University, Department of Electromechanics, Systems and Metal Engineering, Research Group on Dynamical Systems and Control, Technologiepark 125, Gent 9052, East-Flanders, Belgium; b Ghent University Hospital, Department of Anesthesia, Corneel Heymanslaan 10, Gent 9000, East-Flanders, Belgium; c Technical University of Cluj-Napoca, Department of Automation, Memorandumului 28, Cluj-Napoca 400027, Cluj, Romania; d Istanbul Technical University, Department of Control and Automation Engineering, Maslak 34469, Istanbul, Turkey; e National Polytechnic School, Department of Automatics, Advanced Multidisciplinary Industrial & Systems Engineering Laboratory, Rue des Fr‘eres OUDEK 10, El Harrach, 16200, Algiers, Algeria

**Keywords:** Compartmental modelling, General anesthesia, Cole–Cole model, Bioimpedance, Closed-loop control of anesthesia, Obesity

## Abstract

***Introduction*:**

Surgical procedures exceeding six hours and intensive care for traumatic injuries often require prolonged general anesthesia, commonly maintained via induced coma. Similar protocols are used for COVID-19 patients under mechanical ventilation. These conditions pose challenges such as tissue drug accumulation and overdose risk. Extended supine or prone positioning alters tissue volumes based on their physical properties. Accurate anesthesia management depends on patient-specific models that characterize pharmacokinetics (PK, drug distribution) and pharmacodynamics (PD, drug effects) using compartmental representations. However, comorbidities such as obesity are typically overlooked in existing PK models.

***Objectives*:**

This study augments PK models to investigate the impact of obesity on drug distribution and clearance by incorporating the risk of drug trapping in adipose tissue as a nonlinear function of body mass index (BMI). A theoretical framework links BMI with tissue porosity and permeability, introducing a ”trap” compartment to model delayed clearance in obese patients.

***Methods*:**

The model is validated using in vitro impedance measurements and numerical simulations. Fat tissue properties were characterized via Cole–Cole fractional-order models, with parameters identified using a genetic algorithm. The nonlinear BMI–trapping relationship was estimated using the trust region method. Simulations covered both open-loop (single-bolus) and closed-loop (continuous infusion) scenarios using model predictive control(MPC).

***Results*:**

Impedance measurements and identified Cole–Cole fractional-order models confirmed volume-dependent properties of fat tissue. Simulations using clinical data demonstrated delayed drug clearance in high-BMI patients, which support the proposed theoretical background. Open-loop simulations showed prolonged drug retention, while closed-loop control using MPC maintained anesthetic depth with reduced total drug input. Differences in BIS nadir and total drug usage were observed across BMI and age groups, supporting the model’s clinical applicability.

***Conclusion*:**

The augmented model with MPC minimizes drug trapping and reduces total drug use, lowering overdose risk and supporting safer anesthesia management.

## Highlights

• Development of a novel augmented pharmacokinetic model that includes additional fat volume, demonstrating prolonged drug retention as BMI increases.
• Theoretical background for adipose tissue properties: nonlinear relationship between porosity and permeability.
• Modelling a nonlinear function to characterize the risk associated with long tail dynamics of drug trapping in fat tissue.
• Augmented compartment for patient pharmacokinetic model for general anesthesia.
• Design of in vitro experiments and validation of theoretical background with fat samples of different volumes using Cole–Cole fractional order impedance model identification of frequency response
• Numerical validation in 106 patients from clinical trials in response to single bolus (open loop) and reference tracking model based predictive control to maintain general anesthesia (closed loop) illustrating existence and associated effects of drug trapping.

## Graphical abstract

**Figure:**
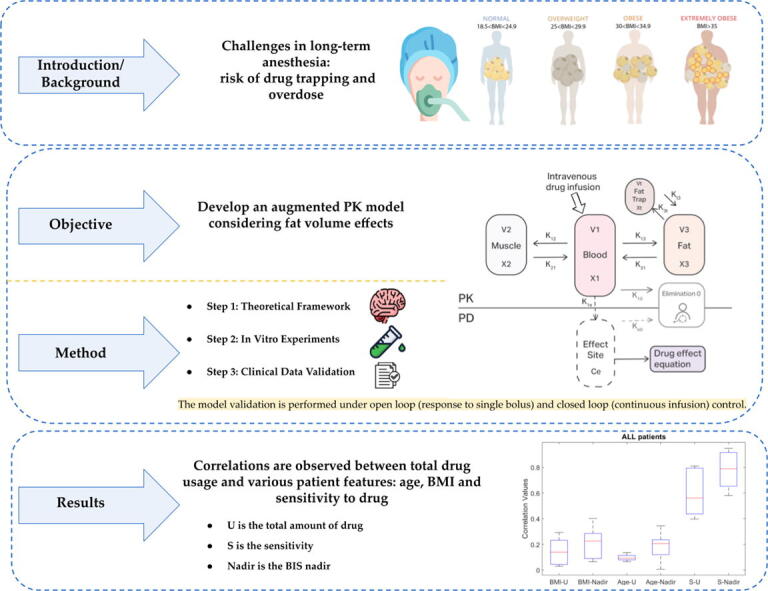

## Introduction

General anesthesia is a drug-induced state characterized by unconsciousness, analgesia, and neuromuscular blockade. It is achieved through the administration of carefully balanced combinations of anesthetics, muscle relaxants, and analgesics to maintain patient safety and comfort during surgery. Certain surgical procedures require prolonged intervention times under long-term general anesthesia in supine position. Indeed, long-term general anesthesia is essential in specific critical situations to stabilize patients following traumatic events and for closely monitoring patients during intensive care [1]. This also occurred in COVID-19 patients placed in the prone position during intensive care treatment for respiratory and hemodynamic management with mechanical ventilation support [2], [3], [4]. In these extreme situations, the compartmental models used to characterize the time constants of drug distribution within the body are no longer valid and need to consider alternate drug diffusion patterns.

Population-based models are widely used to predict the optimal drug dosage for intravenous administration as mentioned in [5]. A pharmacokinetic (PK) compartmental model typically considers compartments representing blood, muscle, and fat [6], followed by the pharmacodynamics (PD) characterizing dose–effect relationship and often described by a nonlinear relationship. Conventional population-based models assume that drugs are distributed homogeneously within soft tissues, muscles, and fat, following a Gaussian distribution with a normal variance [7]. The only distinction lies in their time constants, indeed, the muscle tissue exhibiting faster uptake and clearance rates compared to fat tissue, primarily due to the fact that these tissues have a higher density of blood perfusion. However, population-based models do not take into consideration the effects of comorbidities such as obesity, kidney and/or heart failure, diabetes, etc [8], [3]. Moreover, they typically assume homogeneous drug distribution and fail to capture critical physiological phenomena such as spatial heterogeneity and temporal memory effects. Recent studies have increasingly adopted fractional-order and hybrid models to capture the inherently non-uniform and memory-driven dynamics of biological systems and epidemics. In the context of diabetes modeling, variable-order fractional differential equations have been used to reflect time-dependent memory characteristics of glucose-insulin regulation [9], [10]. Similarly, cancer treatment strategies involving immuno-chemotherapy and gene therapy have been modeled using hybrid fractional operators with time delay [11], [12]. In the study of epidemic diseases such as Ebola and COVID-19, researchers have used piecewise, hybrid, and variable-order fractional operators to capture variable infection rates, memory effects, and intervention strategies [13], [14], [15]. Foundational investigations into fractional partial differential equations in population genetics and biological signaling further validate the use of fractional-order systems in describing nonlinear, spatio-temporal biological phenomena [16], [17], [18]. Collectively, these works highlight a shared focus on non-uniform distribution modeling and the critical role of memory effects in improving the realism and predictive capacity of disease and physiological system models.

Building on this growing line of research, the present study focuses on the pharmacokinetics of general anesthesia, where drug distribution is highly influenced by patient-specific tissue characteristics. In particular, modeling non-uniform diffusion is crucial for capturing the retention of anesthetic agents in adipose tissue, especially in patients with high BMI. While previous studies have addressed non-uniformity in disease dynamics, our study goes beyond the state of the art by applying these principles to anesthesia pharmacokinetics. The novelty of this paper is that it introduces a physiologically grounded, BMI-dependent compartment that accounts for adipose drug trapping during long-term infusion, an aspect rarely considered in anesthesia pharmacokinetics. In this context, biological tissues differ significantly in their structural and transport characteristics, especially during prolonged anesthetic administration. Adipose tissue is notably less perfused, more lipophilic, and structurally heterogeneous, resulting in slower drug uptake and extended retention of anesthetic agents. These variations lead to non-uniform distribution and delayed clearance that are not captured by standard compartmental models based on homogeneous assumptions. In patients with obesity, the increased fat volume and altered tissue composition further accentuate these effects. Accurately modeling such distribution dynamics is critical to improving dosage precision and reducing the risk of accumulation-related complications.

This study addresses the gap in current anesthesia pharmacokinetic models, which typically overlook non-uniform drug diffusion and adipose drug trapping in high-BMI patients, by introducing an augmented PK framework that models non-uniform drug diffusion, specifically the risk of drug trapping in adipose tissue, as a function of BMI. According to a March 2023 report from the World Obesity Atlas, more than half of the global population (51%, or over 4 billion people) will have obesity by 2035, affecting all regions and continents of the world. Obesity significantly increases the risk of various health complications, depending on the procedure carried out [19], which is one of the main comorbidities challenging the surgical results and patient recovery periods [20]. There is also a parallel rise in obese patients admitted to intensive care units (ICU) proportionally with the increase in the global widespread of obesity [21]. In addition, obesity causes a significant increase and changes in adipose tissue, which affects different areas of fat in the body. This can contribute to vascular dysfunction and cardiovascular diseases [22] which introduce additional risks for general anesthesia. Furthermore, evidence shows it affects neuro-cognitive processes in the elderly [23], exacerbates complications and mortality in COVID-19 patients [24], and worsens overall health conditions. Consequently, this contributes to increased complications in patients undergoing general anesthesia [25], [26]. The theoretical framework herein proposed allows us to augment the existing PK patient models with an extra compartment denoting the risk for trapping drug molecules in fat volumes. This includes the effects of comorbidities dependent on the BMI. In vitro examination of fat samples of different volumes supports the theoretical model described herein and encourages its use in management strategies of general anesthesia.

Accurate patient models are essential when managing general anesthesia states as their outcomes influence the risk for over- and under-dosing, leading to critical complications, and affecting the time of recovery to normal vital signs. The criticality thereof is further enhanced by those patients undergoing intensive care traumatic injury and post-traumatic surgery recovery under long term general anesthesia and induced coma states. The patient models assist the anesthesiologist in the decision making process of drug titration. The computer based optimal control algorithm mimicking closely the actions of the anesthesiologist is by its nature a predictive control algorithm. The Model Predictive Control (MPC) is an advanced control methodology that uses a model within the control scheme to forecast the future behaviour of the plant and to optimize future control actions in real-time. This predictive behaviour enables the controller to handle multivariable systems and accommodate constraints imposed by patient safety intervals [27], [28].

The main contributions of present study and the new developments from this article are highlighted and further emphasized as follows:

• Development of a novel augmented pharmacokinetic model that includes additional fat volume, demonstrating prolonged drug retention as BMI increases.
  – Theoretical background for adipose tissue properties: nonlinear relationship between porosity and permeability.
  – Modelling a nonlinear function to characterize the risk associated with long tail dynamics of drug trapping in fat tissue
  – Augmented compartment for patient pharmacokinetic model for general anesthesia.
• Design of in vitro experiments and validation of theoretical background with fat samples of different volumes using Cole–Cole fractional order impedance model identification of frequency response
• Numerical validation in 106 patients from clinical trials in response to single bolus (open loop) and reference tracking model based predictive control to maintain general anesthesia (closed loop) illustrating existence and associated effects of drug trapping.
• Correlations are observed between total drug usage and various patient features such as age, BMI and sensitivity to drug (slope of the dose–effect curve). Significant differences are observed in these correlations when compared with/ without the augmented compartment for drug trap volume as a function of BMI.

The above mentioned contributions are at the core of several disciplines: mathematics, medicine and engineering, the results thereof being significant in all interdisciplinary applications related to these fields.

The remainder of this article is organized as follows. In the next section, we propose the theoretical and experimental background for drug dynamics in fat volumes. Then the augmented PK model is proposed followed by numerical validation of bolus type drug administration to observe the specific long tails of drug trapped molecules in fat tissue and thereby providing the proof of anomalous diffusion existence and further motivation of using fractional order impedance models. Next, we design several numerical simulations and discuss the relevance of the results. Limitations and further developments are summarized in a conclusion section.

## Background

**Fig. 1:**
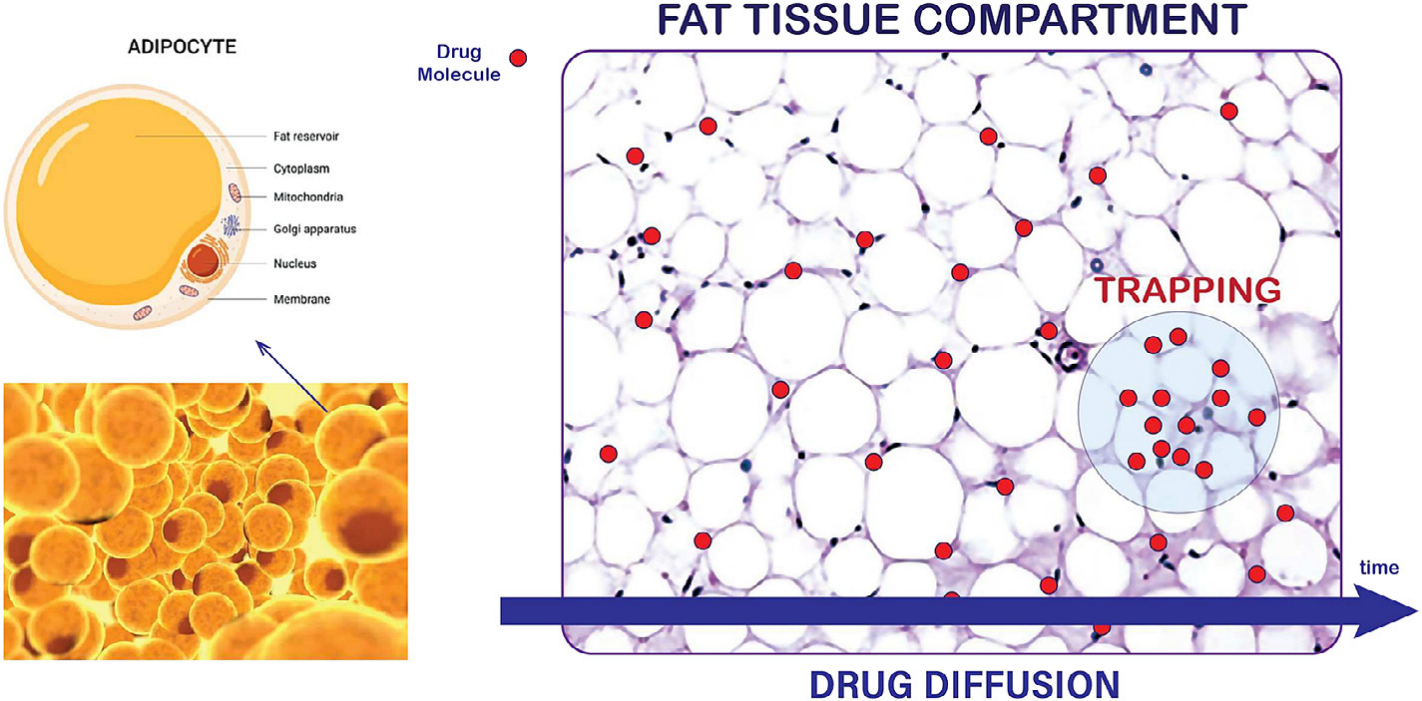
Schematic illustration of the concept of localized drug trapping in fat tissue volume.

Fat tissue, or adipose tissue, is composed of specialized cells called adipocytes, or fat cells, which are responsible for storing and releasing energy. Adipocytes are surrounded by a porous structure that allows diffusion of molecules in and out of the adipose tissue. Fat cells also have a semi-permeable cell membrane that selectively controls the diffusion of molecules. This permeability is crucial for the dynamic processes of molecular transport, enabling the uptake of fatty acids during storage and their release into the bloodstream when energy is needed. The fat tissue contains various types of fat cells whose properties depend on their type or the period of time it has been formed [8]. A schematic representation of the heterogeneous distribution of drug molecules in non–homogeneous fat tissue structure is illustrated in Fig. 1.

**Fig. 2:**
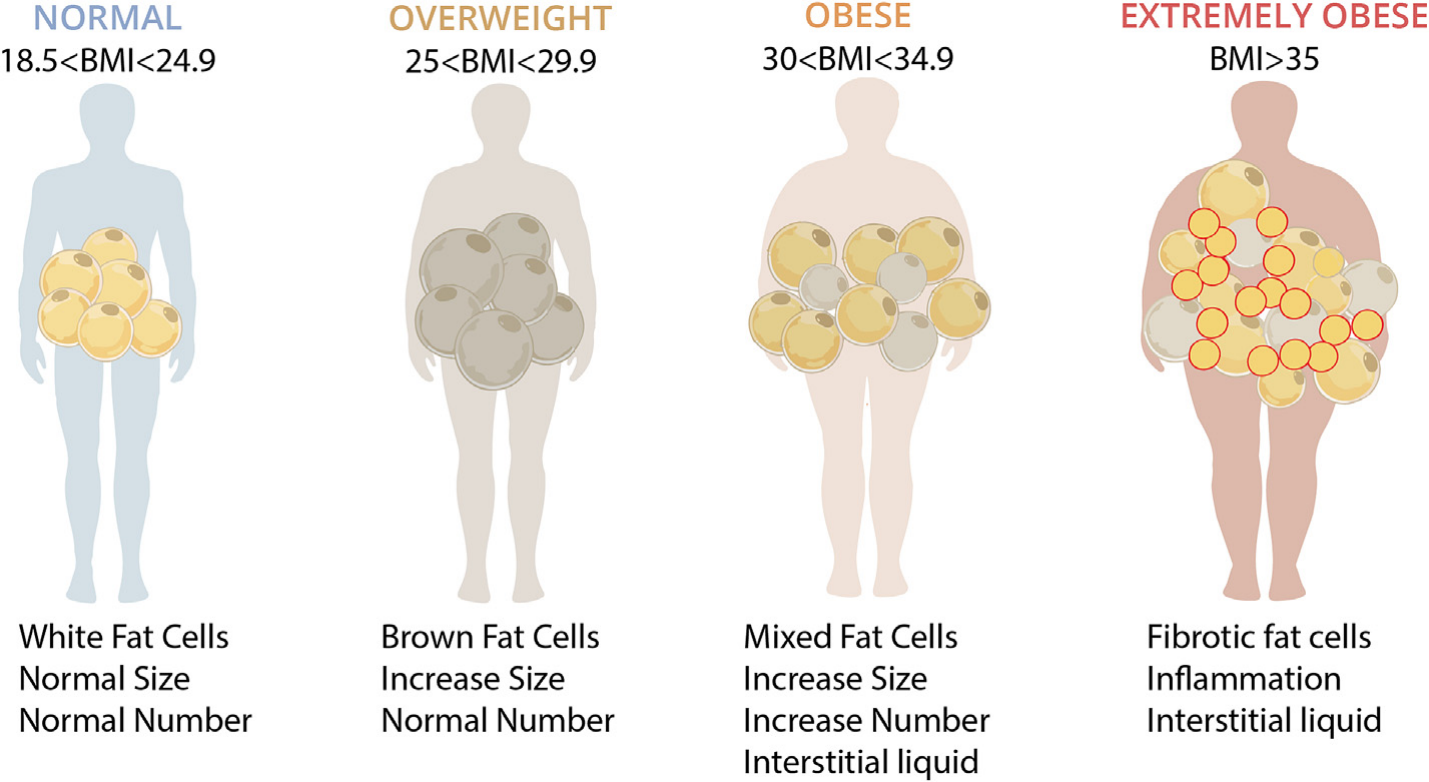
Evolution of fat cells as a function of changes in BMI.

The dynamical stages of fat tissue and their properties can vary depending on BMI, as illustrated in Fig. 2. In individuals with a lower BMI, brown fat, known for its thermogenic properties, is more prevalent. In this case, the balance of interstitial liquid and inner permeability are nominal and can facilitate the molecular binding in-out of the cell, thereby following a normal diffusion pattern as described by Fick’s laws of diffusion [29], [30]. This is often observed in children and lean adults, with normal BMI values. Brown fat generates heat through a process called thermogenesis by breaking down blood sugar (glucose) and fat molecules, aiding in the maintenance of body temperature. A decrease in the body temperature stimulates brown fat, which causes a variety of metabolic alterations that improve permeability through in/out molecular transport of species. Conversely, most of our fat is white fat, which serves as an energy storage depot. Higher BMI individuals often have a greater accumulation of white fat. In this case, the molecular diffusion becomes anomalous at meso- and micro-scales of various lattice matrices of fat tissue structural and their properties [31].

**Fig. 3:**
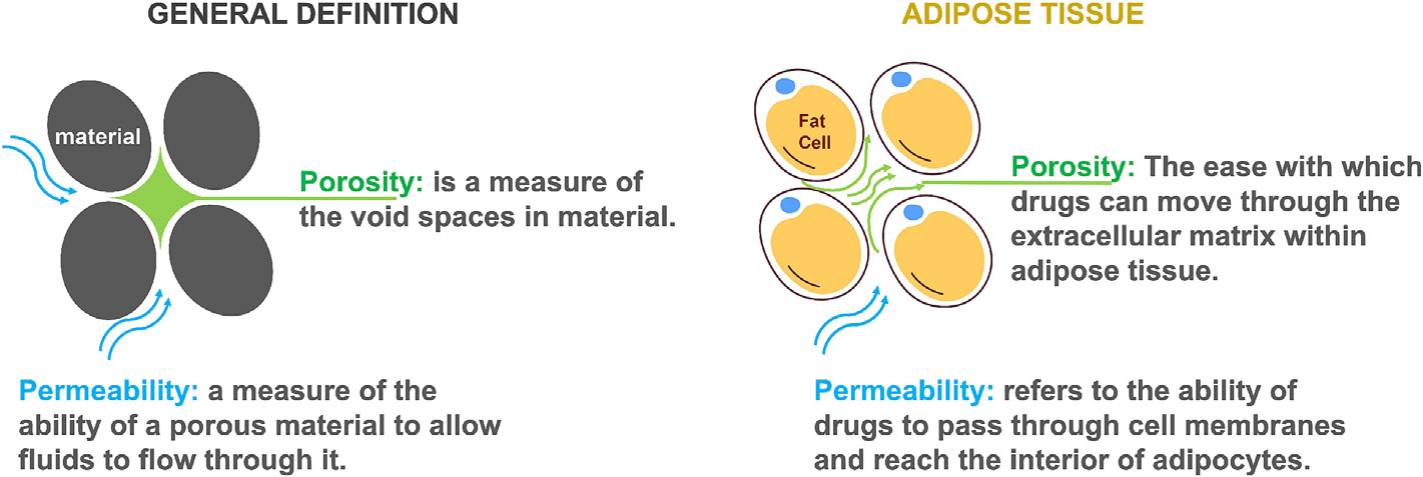
Definition of porosity and permeability: parallel rationale between the general definition and the adipose tissue.

As adipose tissue accumulates and transforms into white fat cells, it aggregates and adheres, increasing in both number and size [32]. Particularly in cases of overweight patients with large BMI values, this leads to significant changes in the dynamics of drug dispersion within the tissue. The increased volume and porosity resulting from fat deposition allow for a greater capacity to transfer drug molecules, but simultaneously, the time constant for dispersion may slow down. This phenomenon highlights the intricate relationship between fat volume, interstitial liquid, and molecule drug diffusion. With continued fat accumulation, the fat cells develop an inflammatory response, leading to fibrosis of the cells and further impaired diffusion [33]. This inflammatory environment not only hinders the diffusion of drugs and reduces angiogenesis in tissue reconstruction but also contributes to the retention of interstitial liquid [34]. Further evidence indicates that local and systemic roles of adipose tissue, along with increased systemic inflammation during obesity, increase their detrimental impact on cardiovascular health [22]. In extreme cases, the porosity pattern becomes mixed whereas the permeability is much impaired, resembling geological composite layers where gravel is mixed with sand and fine sediment, thereby obstructing water flow, Ref. Fig. 3. These complex changes in properties of fat tissue reflect the intricate challenges in drug delivery within the context of obesity.

## Materials and methods

### Defining a comorbidity risk associated with BMI

**Fig. 4:**
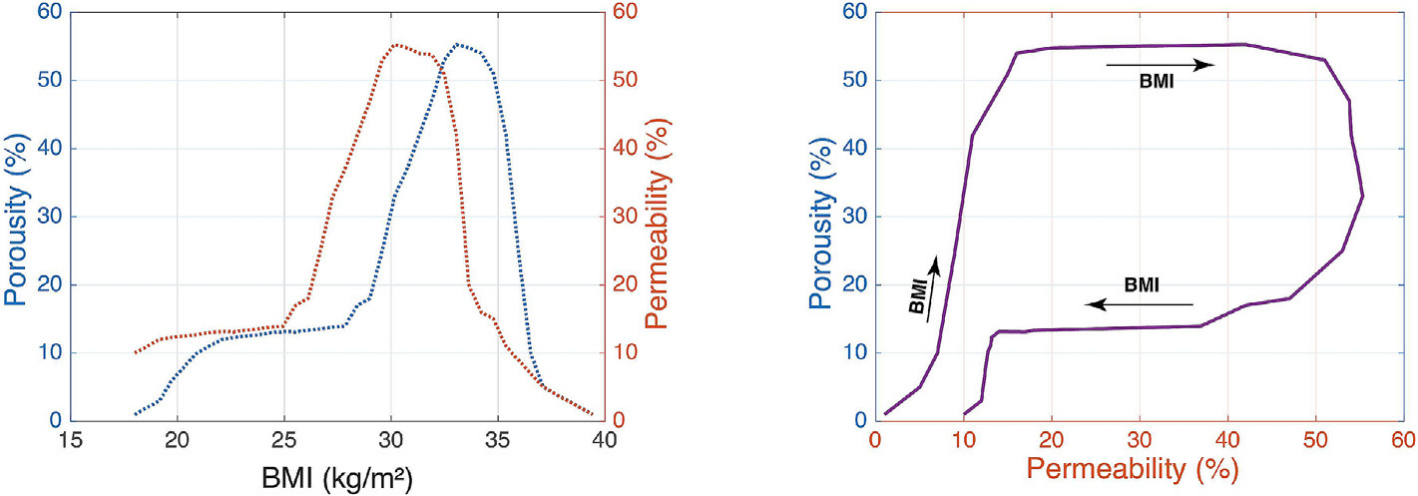
Relation between porosity of fat tissue/permeability of drug molecules and evolution of body mass index (left). Relation between porosity of fat tissue and permeability of drug molecules. Arrows indicate the evolution of BMI from normal to morbidly obese (right).

**Fig. 5:**
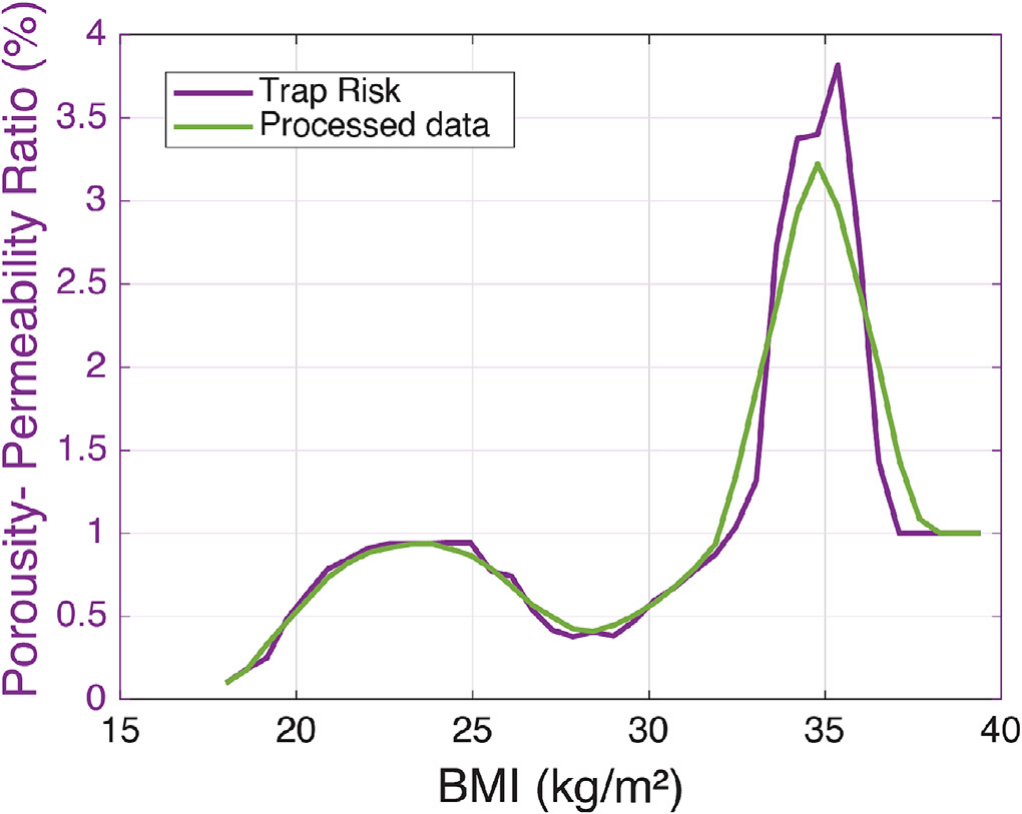
Relation between the relative ratio of porosity to permeability (Trap Risk) against BMI from normal to morbidly obese.

Based on the aforementioned properties, we can observe, as depicted in Fig. 4, the nonlinear relation between the fat tissue porosity function of BMI evolution. The figure represents a bistable equilibrium between the porosity of fat tissue and the corresponding permeability factor of species molecules (e.g. 30% of permeability can have a solution of 10% or 55% porosity). Fig. 5 represents the combined information into a nonlinear relationship between the BMI and the relative ratio of porosity to permeability.

We approximate this relation by a mathematical function using a non-linear least squares method, trust region method [35], in the MATLAB® Mathworks Curve Fitting toolbox. The objective function minimized during the fitting procedure is defined as:

(1) minθ∑i=1n(yi-y^i(θ))2=minθ∑i=1nyi-∑j=1majsin(bj·BMIi+cj)2,

where $y_{i}$ represent data points of the relative ratio of Porosity–Permeability, ${\mathrm{BMI}}_{i}$ is the BMI values corresponding to $y_{i}$. $a_{j},b_{j},c_{j}$ are the parameters of the vector θ (the parameters of multisine model), *n* is the number of data points and *m* is the number of sinusoids used in the model. Further details are given in the Appendix A.

Hereafter, the porosity–permeability ratio as a function of BMI will be denoted as *Risk*, which describes the risk of drug trapping in the adipose tissue.

### Augmented patient model

**Fig. 6:**
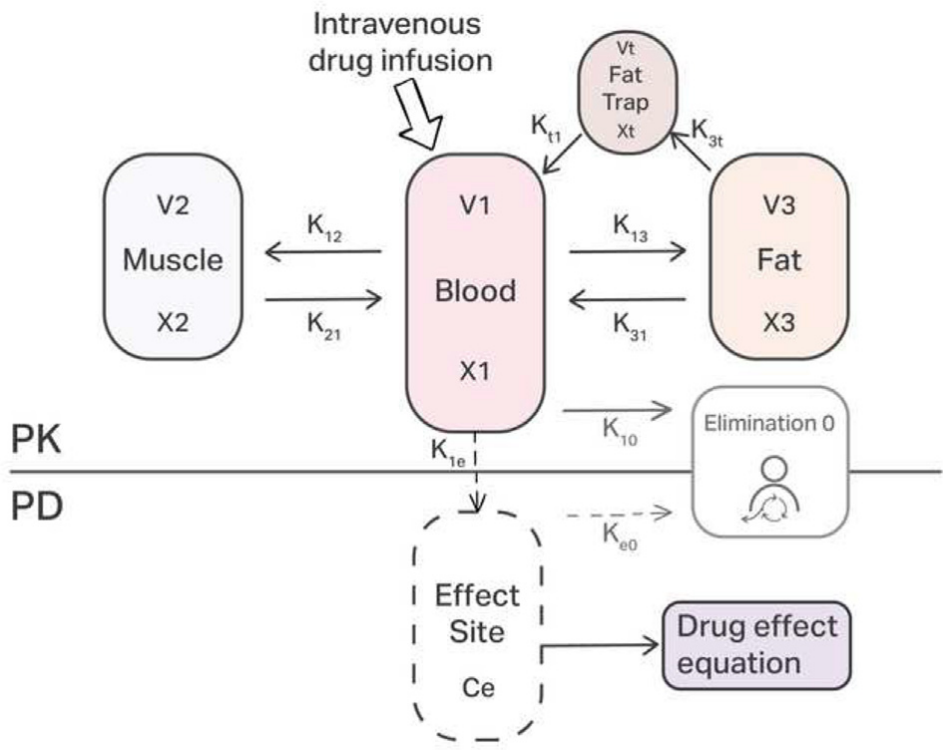
Compartmental model augmented with the additional volume *Vt* of fat cells where drug trapping occurs during long-term general anesthesia.

***Generic PK Model***. In this paper, we consider a three-compartment pharmacokinetic model consisting of a central compartment (blood), a highly perfused compartment (muscle and other vascularized tissues), and a scarcely perfused compartment (fat and poorly vascularized tissues). Three-compartment models are among the most accurate structures for representing drug distribution dynamics [36]. We augmented the classical model by incorporating an additional volume representing fat cells, as depicted in Fig. 6. This study specifically focuses on the Schnider model for Propofol pharmacokinetics, as described in [37].

The differential equations characterizing PK dynamics are given by the relations to the variation of concentrations $x_{i}$ in their respective compartments. For the blood compartment

(2) $${\overset{˙}{x}}_{1}(t)=-k_{12}x_{1}(t)-k_{13}x_{1}(t)-k_{10}x_{1}(t)+k_{21}x_{2}(t)+k_{31}x_{3}(t)+\frac{u(t)}{V_{1}},$$

with u(t) the input infusion rate of drug (Propofol), in [mg/min], and $V_{1}$, in [L], represents the compartmental volume of the blood.

For fast-acting compartment denoting muscle tissue:

(3) $${\overset{˙}{x}}_{2}(t)=k_{12}x_{1}(t)-k_{21}x_{2}(t),$$

and for slow-acting compartment denoting fat tissue:

(4) $${\overset{˙}{x}}_{3}(t)=k_{13}x_{1}(t)-k_{31}x_{3}(t),$$

where the parameters $k_{\mathrm{ij}}$ for i≠j are the drug transfer rates from the $i^{\mathrm{th}}$ to the $j^{\mathrm{th}}$ compartment and they are defined as:

(5) $$k_{10}=\frac{C_{l1}}{V_{1}},k_{12}=\frac{C_{l2}}{V_{1}},k_{13}=\frac{C_{l3}}{V_{1}},k_{21}=\frac{C_{l2}}{V_{2}},k_{31}=\frac{C_{l3}}{V_{3}},$$

where: $V_{1},V_{2}$ and $V_{3}$, in [L], represent the compartmental volume with their clearance rates $C_{l1},C_{l2}$ and $C_{l3}$, in [L/min] respectively:

(6) $$\begin{array}{c} V_{1}=4.27,V_{2}=18.9-0.391\cdot (\mathrm{age}-53),V_{3}=238,\\ C_{l1}=1.89+0.0456\cdot (\mathrm{weight}-77)-0.0681\cdot (\mathrm{lbm}-59),\\ +0.0264\cdot (\mathrm{height}-177),\\ C_{l2}=1.29-0.024\cdot (\mathrm{age}-53),C_{l3}=0.836, \end{array}$$

where *lbm* represents the lean body mass for males:

(7) $$\begin{array}{c} {\mathrm{lbm}}_{m}=1.1\cdot \mathrm{weight}-128\cdot \frac{{\mathrm{weight}}^{2}}{{\mathrm{height}}^{2}}, \end{array}$$

and for females:

(8) $$\begin{array}{c} {\mathrm{lbm}}_{f}=1.07\cdot \mathrm{weight}-148\cdot \frac{{\mathrm{weight}}^{2}}{{\mathrm{height}}^{2}}, \end{array}$$

Typical time constants of these compartments are about 2–5 min for blood compartment, 10–20 min for muscle and larger than 30–60 min for fat compartment.

***Additional PK Compartment***. The proposed additional trap compartment (as depicted in Fig. 6) is given by:

(9) $${\overset{˙}{x}}_{t}(t)=k_{3t}x_{3}(t)-k_{t3}x_{t}(t)\text{.}$$

${\overset{˙}{x}}_{t}(t)$ denotes the concentration of the trapped drug molecules in the fat compartment. $k_{3t}$ and $k_{t3}$ represent the constants of the drug transfer rate from the fat compartment to the fat trap compartment and conversely, with:

(10) $$\begin{array}{c} k_{3t}=\frac{C_{\mathrm{lt}}}{V_{t}},\;k_{t3}=\frac{C_{\mathrm{lt}}}{V_{3}}, \end{array}$$

with its trap volume and clearance rate:

(11) $$V_{t}=V_{3}\cdot \mathrm{BMI}/100,\;C_{\mathrm{lt}}=C_{l3}/\mathrm{Risk}\text{.}$$

*Risk* may be considered as the risk for trapping defined in Table A.6, hence the higher the *Risk* values (as the BMI increases), the slower the clearance from the trap volume. Consequently, the molecules stay longer times in the fat-trap tissue. The BMI represents a numerical value of a person’s weight in relation to their height.

(12) $$\mathrm{BMI}=\mathrm{weight}/{\left(\mathrm{height}\right)}^{2}\text{.}$$

Introducing this new compartment requires to adapt (4) taking into account the new dynamics:

(13) $${\overset{˙}{x}}_{3}(t)=k_{13}x_{1}(t)-k_{31}x_{3}(t)-k_{3t}x_{3}(t)+k_{t3}x_{t}(t)\text{.}$$

***Stability and Convergence***. The dynamics of the three-compartment model can be written in the standard state-space form:

(14) $$\overset{˙}{x}(t)=Ax(t)+\mathrm{Bu}(t)$$

where $x(t)={\left[x_{1}(t),x_{2}(t),x_{3}(t)\right]}^{⊤}$is the state vector, u(t)is the infusion rate of Propofol, and the matrices $A\in R^{3\times 3},B\in R^{3\times 1}$are given by:

(15) $$A=\left[\begin{array}{ccc} -(k_{10}+k_{12}+k_{13}) & k_{21} & k_{31}\\ k_{12} & -k_{21} & 0\\ k_{13} & 0 & -k_{31} \end{array}\right],\;B=\left[\begin{array}{c} \frac{1}{V_{1}}\\ 0\\ 0 \end{array}\right]\text{.}$$

The three-compartment pharmacokinetic model introduced above is a linear time-invariant system characterized by a state matrix $A\in R^{3\times 3}$ whose off-diagonal elements are nonnegative and diagonal elements are strictly negative, due to the presence of inter-compartmental exchange and clearance to the environment. This structure corresponds to a donor-controlled compartmental system, in which each flow depends solely on the state of the donor compartment. Donor-controlled systems are a subclass of compartmental models, where the transfer rates between compartments are driven only by the concentration in the source (donor) compartment, and not by the recipient. This is a standard assumption in pharmacokinetics and physiology, as it reflects realistic mass transfer behavior based on passive diffusion or flow-limited processes. As established in the compartmental modeling literature, such systems are asymptotically stable under mild assumptions. In particular, Brown [38] discusses the structural and dynamical properties of linear compartmental systems with first-order kinetics, showing that positive inter-compartmental and elimination rates ensure physical realizability and boundedness. Sandberg [39] extends this analysis to a broader class of nonlinear systems and proves the existence of a globally asymptotically stable equilibrium under donor-controlled flow conditions and positive outflows. For the linear case, Hearon [40] provides a rigorous stability analysis, demonstrating that when the system matrix has strictly dominant diagonals and the model is open, all eigenvalues lie in the open left half-plane, which guarantees global exponential stability. Based on this result, we can safely assume that the dynamics of the three-compartment model are globally asymptotically stable, and all state trajectories converge to a unique equilibrium as t→∞.

Given the established stability of the three-compartment model, it is of interest to investigate whether this property is preserved after adding the trap compartment that accounts for drug retention in adipose tissue. To assess the internal stability of the patient-specific PK model, we consider the state-space system defined by

$$\overset{˙}{x}(t)=\mathrm{Ax}(t)+\mathrm{Bu}(t),\;y(t)=\mathrm{Cx}(t),$$

where the input u(t)represents drug administration rate, and the output y(t)is the predicted central compartment concentration. The system matrices are given by

$$A=\left[\begin{array}{cccc} -(k_{12}+k_{13}+k_{10}) & k_{21} & k_{31} & 0\\ k_{12} & -k_{21} & 0 & 0\\ k_{13} & 0 & -(k_{31}+k_{3t}) & k_{t3}\\ 0 & 0 & k_{3t} & -k_{t3} \end{array}\right],\;B=\left[\begin{array}{c} \frac{1}{V_{1}}\\ 0\\ 0\\ 0 \end{array}\right],$$

$$C=\left[\begin{array}{cccc} 1 & 0 & 0 & 0 \end{array}\right],\;D=0\text{.}$$

The characteristic polynomial of the system is given by

(16) $$\det(\mathrm{sI}-A)=a_{4}s^{4}+a_{3}s^{3}+a_{2}s^{2}+a_{1}s+a_{0},$$

with coefficients expressed symbolically as

(17) $$\begin{array}{cc} & a_{4}=1,\\ & a_{3}=k_{10}+k_{12}+k_{13}+k_{21}+k_{31}+k_{3t}+k_{t3},\\ & a_{2}=k_{10}k_{21}+k_{13}k_{21}+k_{10}k_{31}+k_{12}k_{31}+k_{21}k_{31}+k_{10}k_{3t}+k_{12}k_{3t}\\ & \;+k_{13}k_{3t}+k_{21}k_{3t}+k_{10}k_{t3}+k_{12}k_{t3}+k_{13}k_{t3}+k_{21}k_{t3}+k_{31}k_{t3},\\ & a_{1}=k_{10}k_{21}k_{31}+k_{10}k_{21}k_{3t}+k_{13}k_{21}k_{3t}+k_{10}k_{21}k_{t3}+k_{13}k_{21}k_{t3}\\ & \;+k_{10}k_{31}k_{t3}+k_{12}k_{31}k_{t3}+k_{21}k_{31}k_{t3},\\ & a_{0}=k_{10}k_{21}k_{31}k_{t3}\text{.} \end{array}$$

**Fig. 7:**
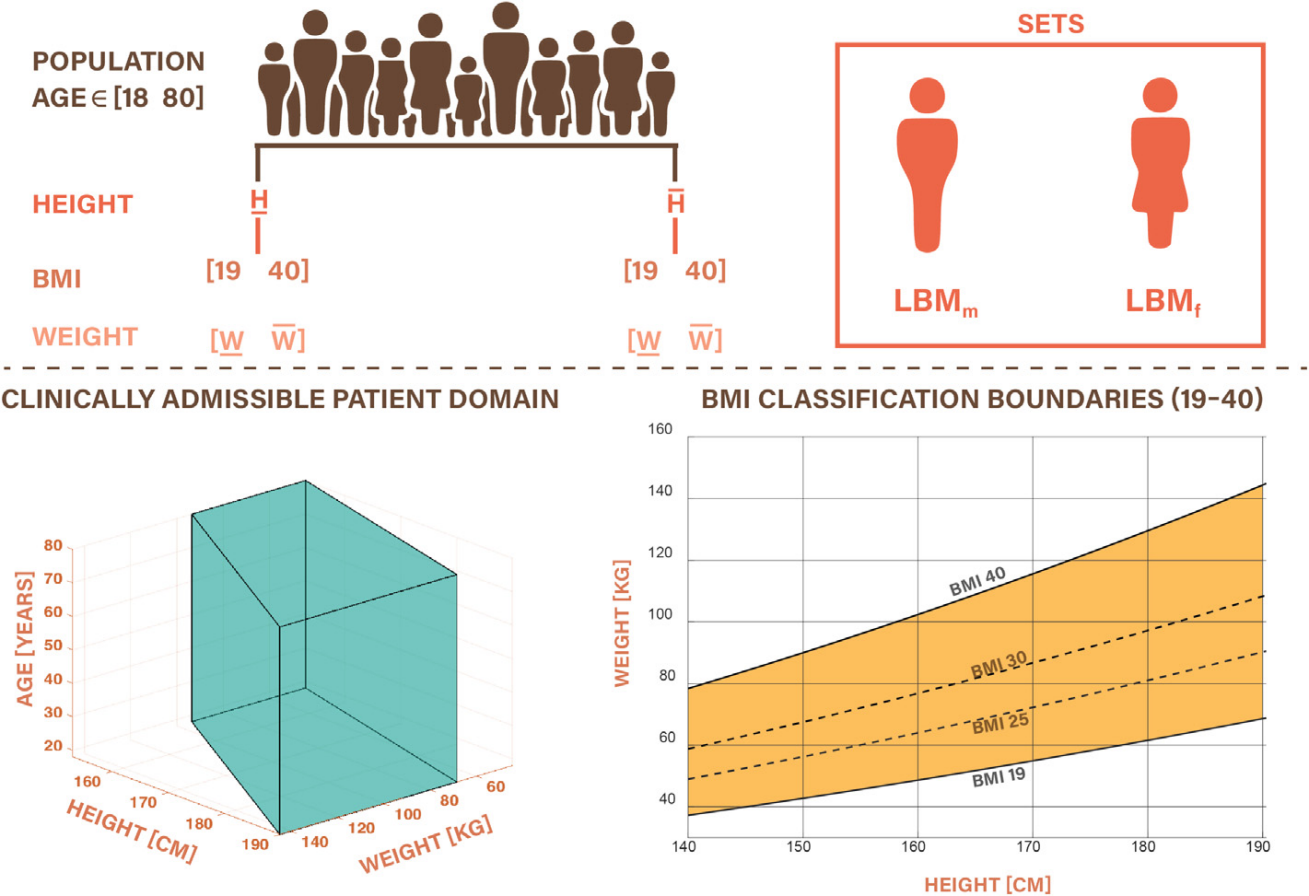
Definition of the clinically relevant input domain for model parameterization. The European adult population is stratified by gender, age [18,80] years, and height (female: 150–190 cm). For each height, the admissible weight range is determined to ensure the BMI remains within the physiological range [19,40]. The resulting domain is visualized as overlapping 3D boxes in (height, weight, age) space.

The parameters $k_{\mathrm{ij}}$ are physiological rate constants defined from patient-specific variables (age, height, weight, BMI) through known PK formulas, as previously introduced in Eqs. (5), (10), including nonlinear expressions for clearance and lean body mass in (6), (7), (8), (11). Due to variability in age, height, and weight, the coefficients $a_{i}$ are not fixed but vary within bounded intervals. For the clinically relevant adult population, we defined the input domain as:

(18) age∈[18,80]years,BMI∈[19,40],height∈[150,190]cm,

with weight bounds at each height chosen to satisfy the BMI constraints. Using these ranges, we enumerated all extreme combinations (8 geometric corner patients as shown in Fig. 7 bottom left) and included additional patients corresponding to the BMI values where the function (13) attains its global minimum and maximum within the interval of BMI [19], [40].

The internal stability of the augmented four-compartment model was assessed using Kharitonov’s theorem and the Routh-Hurwitz criterion, as detailed in Appendix C. By constructing interval polynomials from the parameter bounds defined over the clinically admissible population domain in Fig. 7, stability verification was reduced to testing a finite set of extreme cases. For both genders, all four Kharitonov polynomials were confirmed to be Hurwitz. Therefore, the entire family of patient-specific PK models is guaranteed to be internally stable across the full range of age, height, weight, and BMI considered in this study.

***Generic PD Model***. In many cases, modeling the response solely on systemic concentrations in the PK model is insufficient. This issue arises particularly when there is a delay between a delay between the peak plasma concentration and the peak pharmacodynamic effect [41]. To address this, an additional compartment, known as the effect site compartment, is incorporated into the model to represent the tissue near the target cells. This compartment corresponds to the linear part of the PD model, with a rate for Propofol equal to $k_{e0}=k_{1e}=0.456$. The effect site compartment, which characterizes the drug action in the brain leading to hypnosis (loss of awareness), is given by the first-order equation:

(19) $${\overset{˙}{x}}_{e}(t)=k_{1e}x_{1}(t)-k_{e0}x_{e}(t),$$

with the effect site concentration $C_{e}$ in [mg/L] used to calculate the dose–effect nonlinear relationship, described as the Hill function:

(20) $$E(t)=E_{0}-E_{\max}\frac{x_{e}^{\gamma }(t)}{x_{e}^{\gamma }(t)+C_{50}^{\gamma }},$$

where $C_{50}$, in [mg/L], is the concentration at half effect (50%), γ [-] describes the steepness of the concentration-effect relationship, $E_{0}$ is baseline effect (no drug) and $E_{\max}$ is maximum possible effect. Apart from the induction phase where the nonlinear sigmoid shape of the function from (20) is traversed, the maintenance of anesthesia at a constant depth (around half effect) can be approximated by a linear function [42]. Therefore, during long-term anesthesia, Eq. (20) can be reduced to a linear approximation as a mere gain scheduling control strategy.

Relation (20) is measured by commercial monitors as Bispectral Index with values from 0 (isoline) to 100 (full awake patient). An analysis of this nonlinear function and relationship to exponential-logarithmic functions has been given in [43].

The implementation of this model in Matlab Mathworks is given in the Supplementary Material file.

### Fat sample tissue: in vitro experiments and model identification

**Fig. 8:**
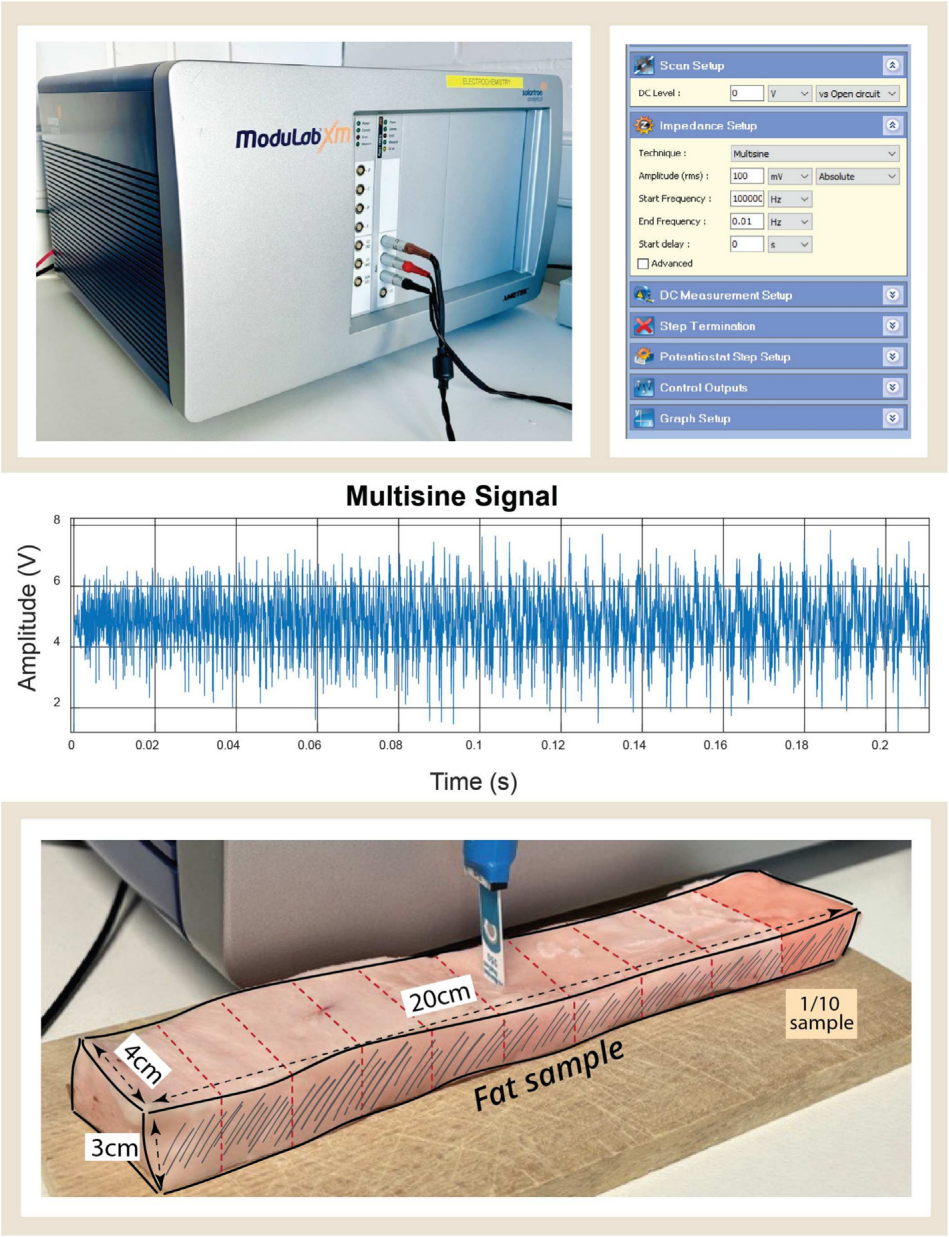
Top: The Solartron spectroscopy device with the input (multisine) specification. Middle: the multisine signal input. Bottom: the ceramic sensor in the fat tissue sample and its dimension.

***Experiments***. Fat samples of various volumes were tested in order to investigate how dynamic impedance properties vary as a function of volume [44]. For this, we use an animal fat tissue sample of 20 cm in length, 4 cm in width, and 3 cm in height and weight of 50 g. The sample was tested using the *Modulab XM* device over a wide spectrum of frequency in high-resolution steps as depicted in Fig. 8.

**Fig. 9:**
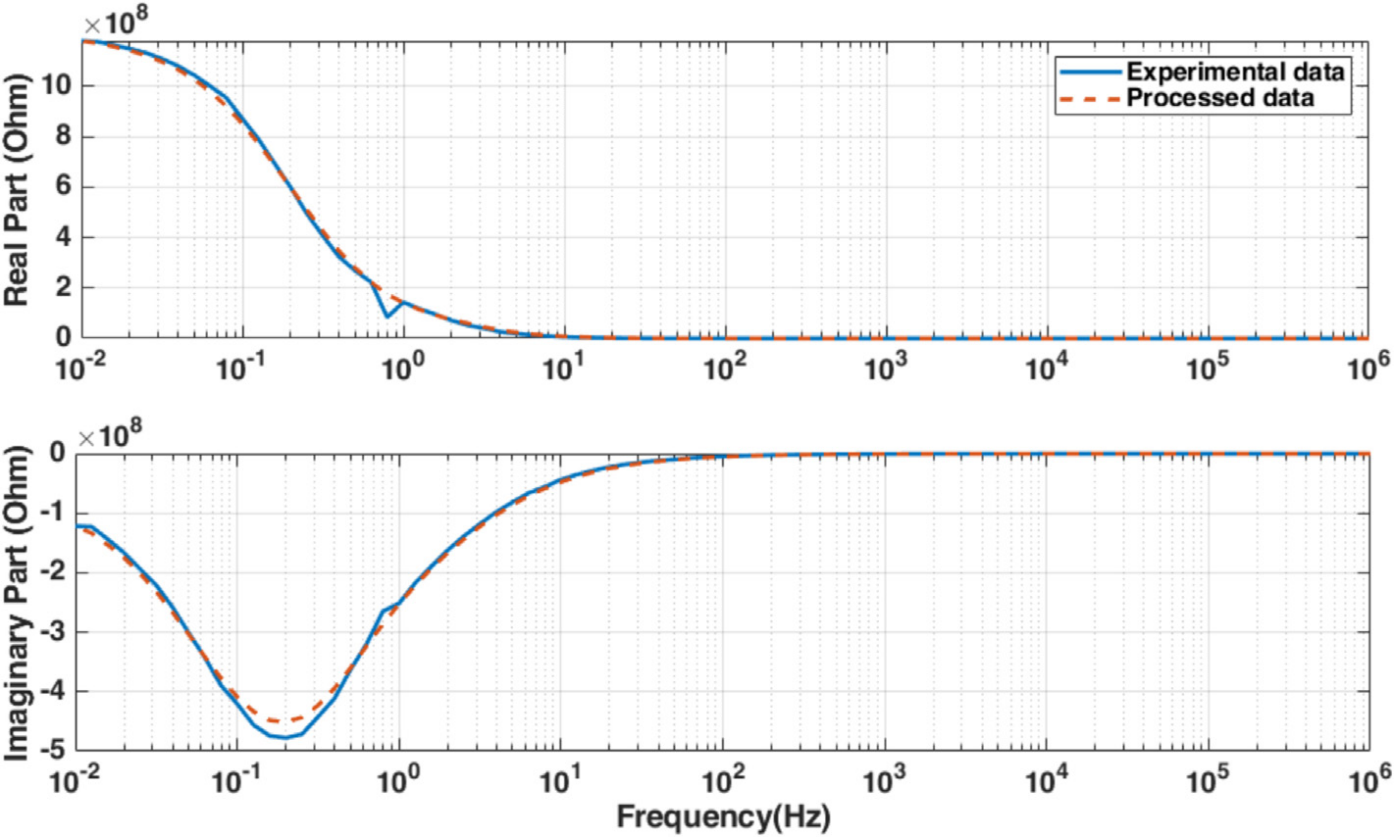
Complex impedance data from one fat tissue sample (2/10) as a function of frequency. The data was smoothed to eliminate noise before the identification step.

The first step of the protocol was to apply a reference signal in the frequency ranges $10^{-2}$ to $10^{3}$ Hz in 100 frequency points ($N_{s}=100$). The measured current and voltage are used to calculate the sample’s impedance (*Z*). The data was recorded for further processing as real (Re(Z)) and imaginary (Im(Z)) parts of complex impedance as a function of test frequency, depicted in Fig. 9. The protocol for testing the fat sample tissue starts with the sample having its maximum volume. Subsequently, for each measurement, one-tenth of the full sample is removed, and the protocol is repeated. This process continues through 10 iterations, each time removing one-tenth of the sample until 1/10 of the original sample remains. The data collected is then used as our population sample for parametric model identification. Further values of data samples are given in the Supplementary Material file.

***Model Structure and Identification***. The Cole–Cole model is the natural model structure for characterizing biological tissue impedance data [45], [46], [47] and has been used successfully to characterize biological samples [48], [49], [50], [51]. It is a mathematical representation used to describe the frequency-dependent electrical response of complex systems, particularly in dielectric spectroscopy [52], [51]. The model incorporates a complex impedance function that accounts for both the distribution of relaxation times and the dispersion of relaxation processes within a material [53], [54]. The lumped fractional order impedance model (FOIM) is then:

(21) $$M_{\mathrm{FOIM}}(s)=R+\frac{K}{1+{\left(\frac{s}{P}\right)}^{\beta }},$$

where *R* denotes the high-frequency resistance, *K* is the magnitude of the dispersion, *P* is the characteristic frequency (in rad/s) that defines the onset of dispersion effects, and β∈(0,1) is the dispersion parameter. A value of β=1 corresponds to Debye behavior, representing a single relaxation time, while β<1 indicates a distribution of relaxation times. By analogy with Debye-type materials, the relaxation time constant can be defined as τ=1/P. The gain parameter *K* determines the magnitude of the impedance variation between low and high frequencies, while *P* and β govern the frequency-dependent shape of the response [47].

**Fig. 10:**
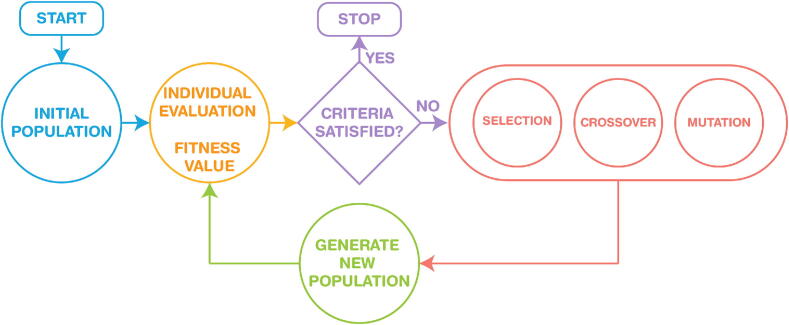
Set of steps within a genetic algorithm iterative optimum search.

Given the complexity and the nonlinearity of this model, a genetic algorithm (GA) optimization has been chosen for searching optimality of the cost function, with the function in Matlab ga in [55]. GA is a heuristic search and optimization technique inspired by the process of natural selection. It mimics the principles of biological evolution to iteratively generate and improve solutions to optimization and search problems. The algorithm solves stochastic global search optimization problems by repeatedly modifying a population of individual solutions and has the following iterative optimum search steps illustrated in Fig. 10.

The algorithm generates a random population of the FOIM model parameters as an initial population:

(22) Z^(jω)=R∗+K∗1+(jωp∗)β∗=Re(Z^)+jIm(Z^),

while a fitness function calculates the difference between measured impedances (*Z*) and the impedances predicted by the model for each parameter (Z^) set:

(23) ERe=1Ns∑i=1100(Re(Z)-Re(Z^))2EIm=1Ns∑i=1100(Im(Z)-Im(Z^)2Error=ERe2+EIm2.

Through selection, crossover, and mutation, the next children generation is produced at each step by selecting individuals from the current population with parameters that better fit our data to be parents. Over successive generations of elite retention, the population of parameter sets converges towards an optimal or near-optimal solution until stopping conditions are met, giving a set of parameters for the FOIM $\left(R^{*},K^{*},P^{*},\beta^{*}\right)$ that best fits the experimental impedance data with the lowest error [56], [57].

### Ethics statement

The data for the numerical simulations was collected according to ethical policies as follows:

For the database of 24 patients, the clinical related information can be found on ClinicalTrials.gov/NCT00735631, and was compliant with the regulatory framework stated in the European Regulation (EU) 2017/745, and approved by the Ethics Committee of Ghent University Hospital, Belgium.

For the 70 patients database, the clinical investigation involving human subjects was compliant with the regulatory framework stated in the European Regulation (EU) 2017/745. This academic clinical investigation was approved by the Ethics Committee of Ghent University Hospital and the Federal Agency for Medicines and Health Products of Belgium FAGG (EC/BC-08020, FAGG/80M0840, EudraCT: CIV-BE-20–07-0342442020, clinicaltrials.gov: NCT04986163).

### Patient database for numerical simulations

For the purpose of numerical analysis of model outcomes, we use patient PK-PD model characteristics as follows:

• A database of 12 virtual patients, representing a wide range of patient specific PK dynamics, as published in [58];
• A database of 24 real patients, with a relatively high age distribution affecting PK model dynamics, as published in [42], [59].
• A database of 70 real patients, with a broad range of patient specific PK model dynamics, as published in [49], [60].

Details of patient characteristics used in the PK-PD model are given in Appendix B.

**Fig. 11:**
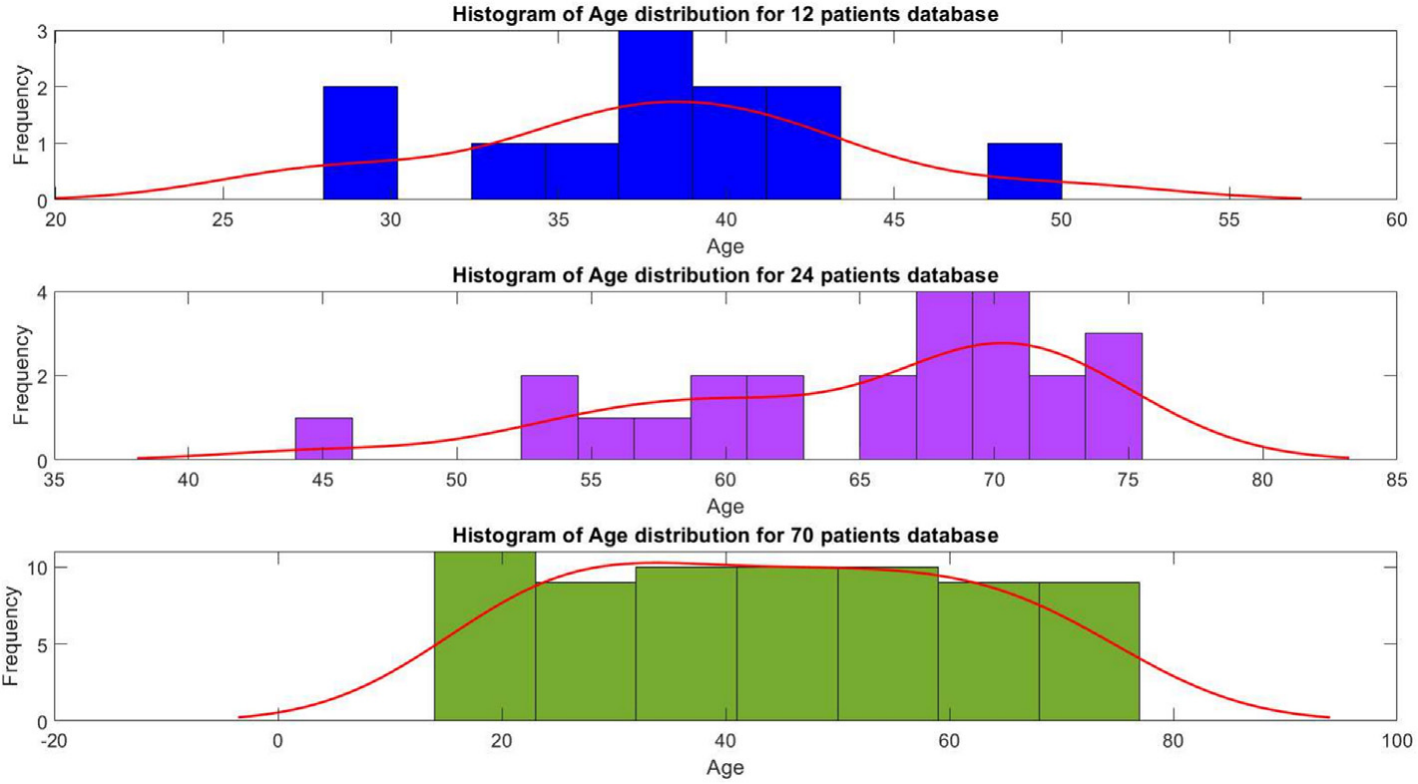
Histogram of Age values distribution in three patient databases used in this study.

**Fig. 12:**
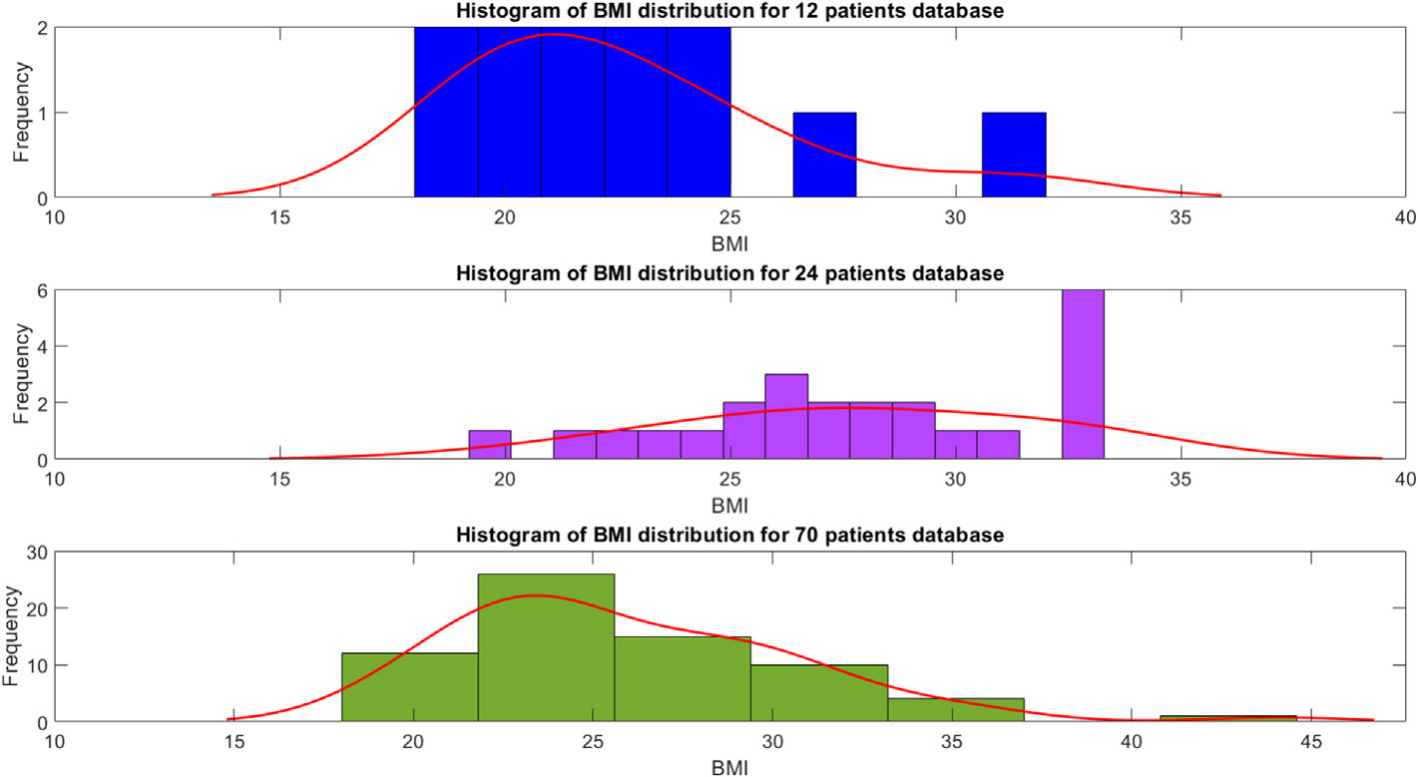
Histogram of BMI values distribution in three patient databases used in this study.

**Fig. 13:**
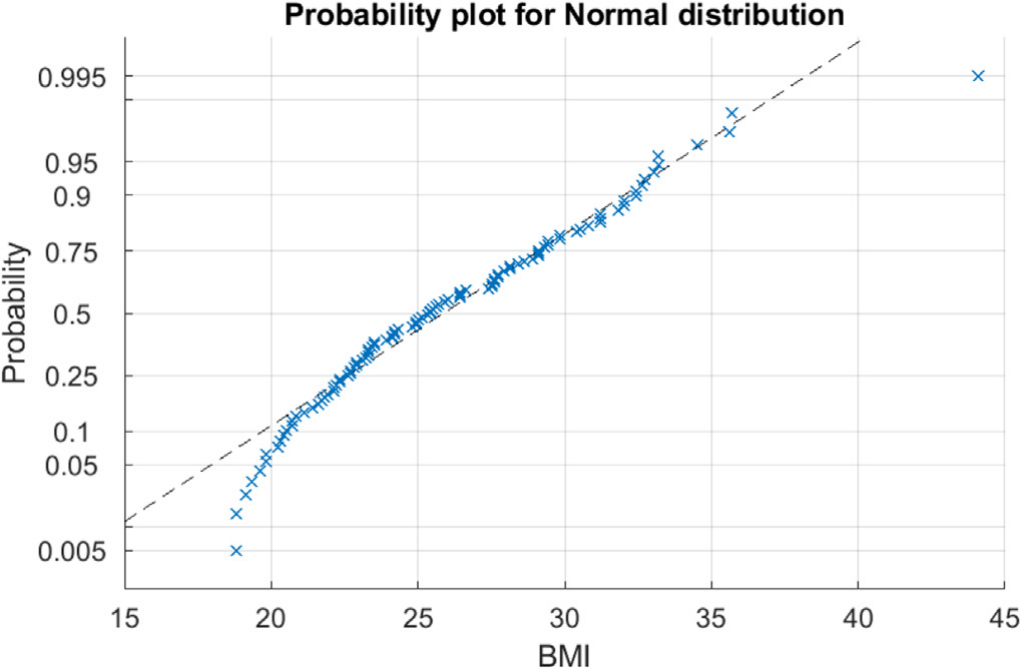
Probability plot of the BMI for all patients in the 3 datasets from Tables B.7, B.8, and B.9.

The corresponding PK model coefficients distributions of these three patient databases are given for age in Fig. 11 and for BMI in Fig. 12 and Table 1. The figure indicates that the majority of the population falls within a BMI range of 20 to 34, spanning from normal to obese, a smaller portion is either underweight or extremely obese. This suggests a normal distribution within the population. This hypothesis is confirmed with the Probability plot in Fig. 13. Patients indexed 1, 41, 63, and 6 from the 70 patient database are considered outliers in this study, as it excludes individuals who are underweight or have a BMI above 40. (see Table 2, Table 3).

**Table 1:** Classification of the patients based on their BMI.

Table	Underweight	Normal Weight	Overweight	Obese	Extremely Obese
12 Patients	0	10	1	1	0
24 Patients	0	6	11	7	0
70 Patients	3	32	23	9	3
Total	3	48	35	17	3

**Table 2:** Category of the patients divided according to their BMI.

	Normal	Overweight	Obese	Extremely Obese
Interval of BMI	18.5–24.9	25–29.9	30–34.9	>35

**Table 3:** Category of the Patients divided according to their AGE.

	Young adults	Middle aged adults	Older adults
Interval of Age	18–35	36–53	54–74

**Fig. 14:**
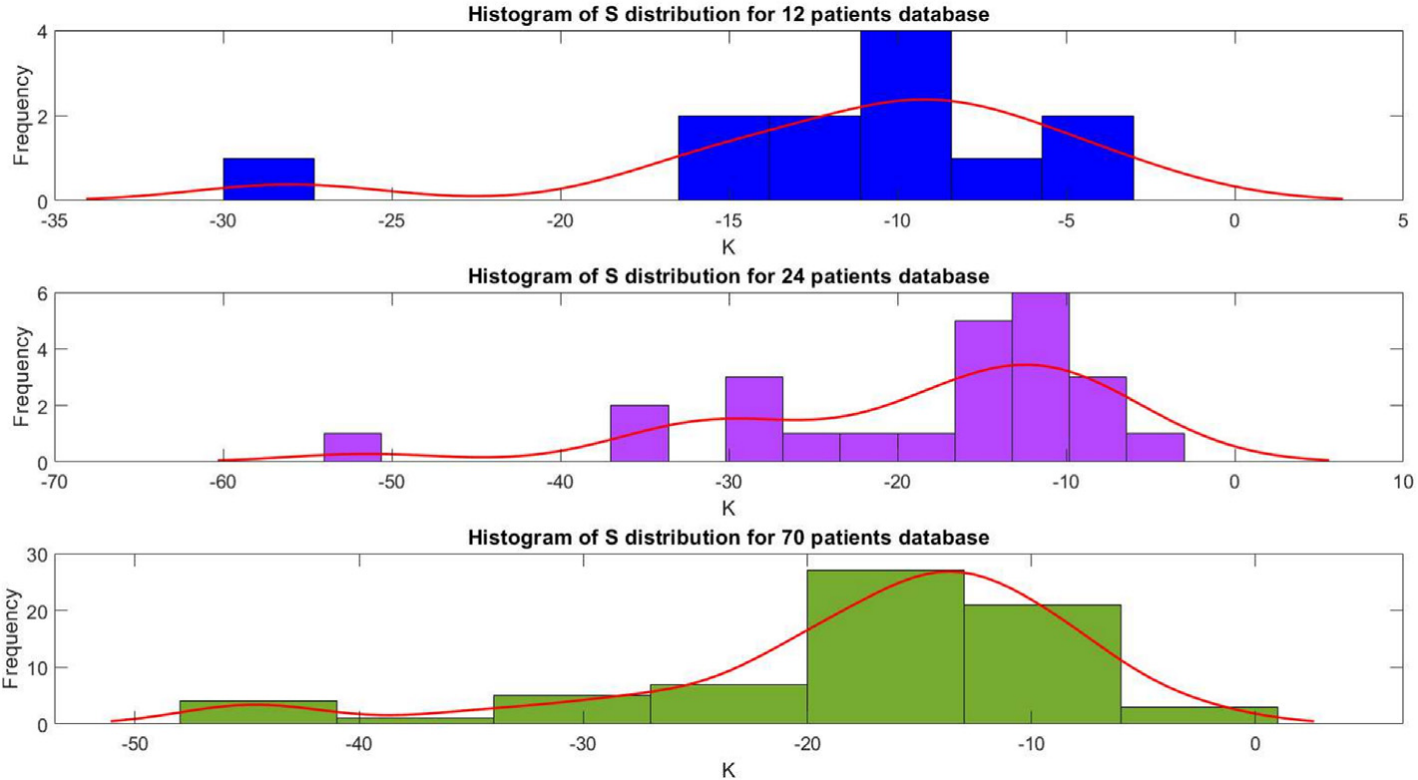
Histogram of Gain *S* (sensitivity) distribution in three patient databases used in this study.

An important PD model dynamic characteristic is the gain of the dose–effect relationship, essentially denoting the patient’s sensitivity to the drug effect. This sensitivity can be determined analytically as described in [61] when the Eq. (20) is linearised around the desired BIS value of 50, the sensitivity can be given as:

(24) $$S=\frac{-\gamma }{C_{50}}*\frac{\left(E_{\max}-E_{0}+50)*(E_{0}-50\right)}{E_{\max}}$$

and the corresponding distributions of this gain in the three patient databases are given in Fig. 14.

### Model based predictive control of general anesthesia

When safety is of primary importance, there are specific solutions at hand for optimization based on available and incoming information to controllers, as well as predicted probability of safety assurance limits [62], [63], [64], [65]. As the closest candidate to mimick actual anesthesiologists decisions, model based predictive control is a successful and mature technology breaking through into medical applications [27], [66]. Developed in early 80s, the extended prediction self-adaptive control (EPSAC) is a generalized form of input–output formulation of predictive control [67], [68]. EPSAC has been successfully applied in control of anesthesia in simulations [58] and in clinical studies [69].

**Fig. 15:**
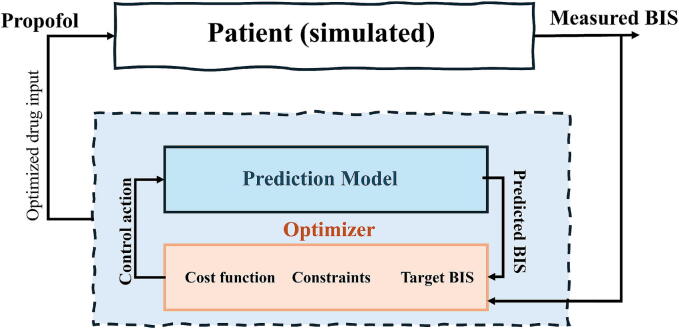
An example of predictive control strategy with patient simulator (Real model) and predictor model (Averaged patient model), along with other data flows for the optmization algorithm.

An illustrative example of block diagram of EPSAC predictive control is given in Fig. 15. While maintaining anesthesia, the computer uses an optimization algorithm to search for optimal drug infusion values for reference BIS tracking, with an automatic decision process but under the supervisory actions of the anesthesiologists. Patient models provide information sources to the optimizer, which uses models to predict and optimize the input according to interval constraints for patient safety. If real-time identification of the actual patient is performed, individual patient models are provided, such as those published in [49], [70]; otherwise, population-based averaged patient models are used instead [71]. Ideally, the patient models are as accurate as possible for the individual patient response and specific surgery performed as to lead to a digital twin of the real patient [72]. If the real process and the prediction model are identical, then we are in the *ideal case* scenario, i.e. best achievable performance in a global optimum sense. Having any differences between these case scenarios will introduce changes in performance from the modelling errors between the real process and the prediction model. In this paper, we introduce a singular error source as with or without the fat trap volume model. In this way, we can safely examine its effects in the closed-loop performance.

As performance measures we introduce:

• The total amount of drug used for each patient (total input)
  (25) $$\begin{array}{c} U_{x}(j)=\overset{240}{\underset{i=1}{\sum }}U_{\mathrm{Prop}\_\mathrm{Cx}}(i,j)\cdot T_{s}\\ \;j=1,\ldots ,\mathrm{total}\;\mathrm{number}\;\mathrm{of}\;\mathrm{patients},\;x=1,2,3,4 \end{array}$$
• The BIS nadir for each patient (Minimum output)
  (26) $$\begin{array}{c} {\mathrm{nadir}}_{x}(j)=\min(Y_{\mathrm{BIS}\_\mathrm{Cx}}(:,j))\\ \;j=1,\ldots ,\mathrm{total}\;\mathrm{number}\;\mathrm{of}\;\mathrm{patients},\;x=1,2,3,4 \end{array}$$

where *x* represents the case number (that will be introduced in the Result section), $U_{\mathrm{Prop}\_\mathrm{Cx}}(i,j)$ represents the amount of drug infused at the sample *i* for patient $j,Y_{\mathrm{BIS}\_\mathrm{Cx}}(:,j)$ represent the output vector for patient *j*.

Tuning predictive control algorithms has been extensively described in [28], [27] and in particular, a user friendly tuning of EPSAC parameters has been described in [73] for many processes both in simulated and experimental conditions.

### Statistical analysis methods

The statistical analysis of the data was conducted using various tools to ensure a comprehensive evaluation of the results. One-way analysis of variance (ANOVA) was performed using the MATLAB® function anova1, which provides a boxplot representation of the median, quartiles, and outliers for groups of samples assumed to follow a normal distribution. To further assess pairwise differences between group means, post hoc analysis was conducted using the Tukey–Kramer method. The correlation coefficient, calculated using the function corrcoef, was employed to determine the similarity between signals. Additionally, quantile–quantile (QQ) plots were generated to visually inspect the normality of the data distributions, providing a comparison between the sample quantiles and the theoretical quantiles from a normal distribution. All these tools were used from the Statistics and Machine Learning Toolbox® in MATLAB®.

## Results

The three patient databases are used in this section to examine various aspects of models properties such as risk and drug trapping volume concentrations, and of maintenance performance of general anesthesia. The detailed biometric values are given in corresponding tables in Appendix B: Table B.7 for the virtual 12 patients, Table B.8 for the real elderly patients and Table B.9 for the real patients with wide range of age distribution.

### Identification of nonlinear risk function from patient database

**Fig. 16:**
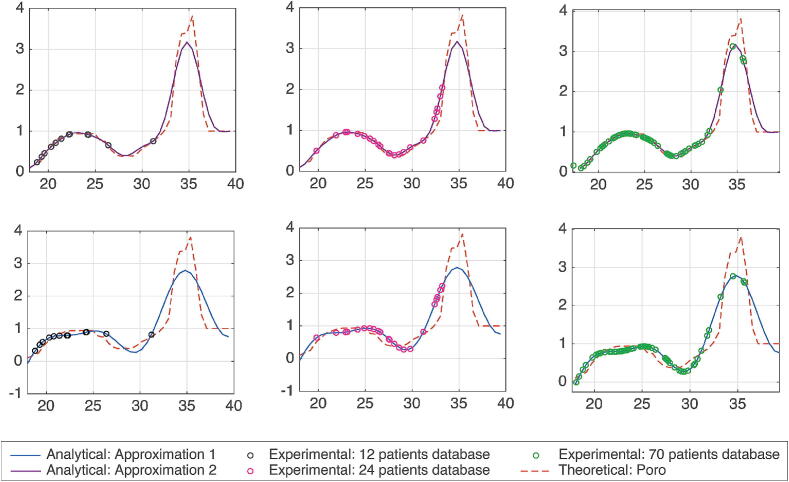
Relation between the *Risk* (y-axes) against BMI (x-axes) from normal to morbidly obese. The continuous line plots - represent the two approximations of Table A.6: Blue (second line) is the first approximation - purple (first line) is the second approximation. The dashed plot – is the theoretical data from Fig. 5. and the circles represents the experimental data: The first column are patients from Table B.7, the second column are patients from Table B.8 and the third column are patients. from Table B.9. (For interpretation of the references to colour in this figure legend, the reader is referred to the web version of this article.)

Here we provide the values of risk of drug trapping using the BMI of these patients and the equations represented in Table A.6. Fig. 16 displays the analytical, the theoretical, and the experimental results of the *Risk* of drug trapping in the proposed fat volume trap compartment.

For the simulation, the Risk in 11 is calculated using the first approximation in the Table A.6 which is a 3 sum of sine equation. The latter is a good compromise between complexity and accuracy, hence it will used hereafter.

### Fat sample model identification

**Fig. 17:**
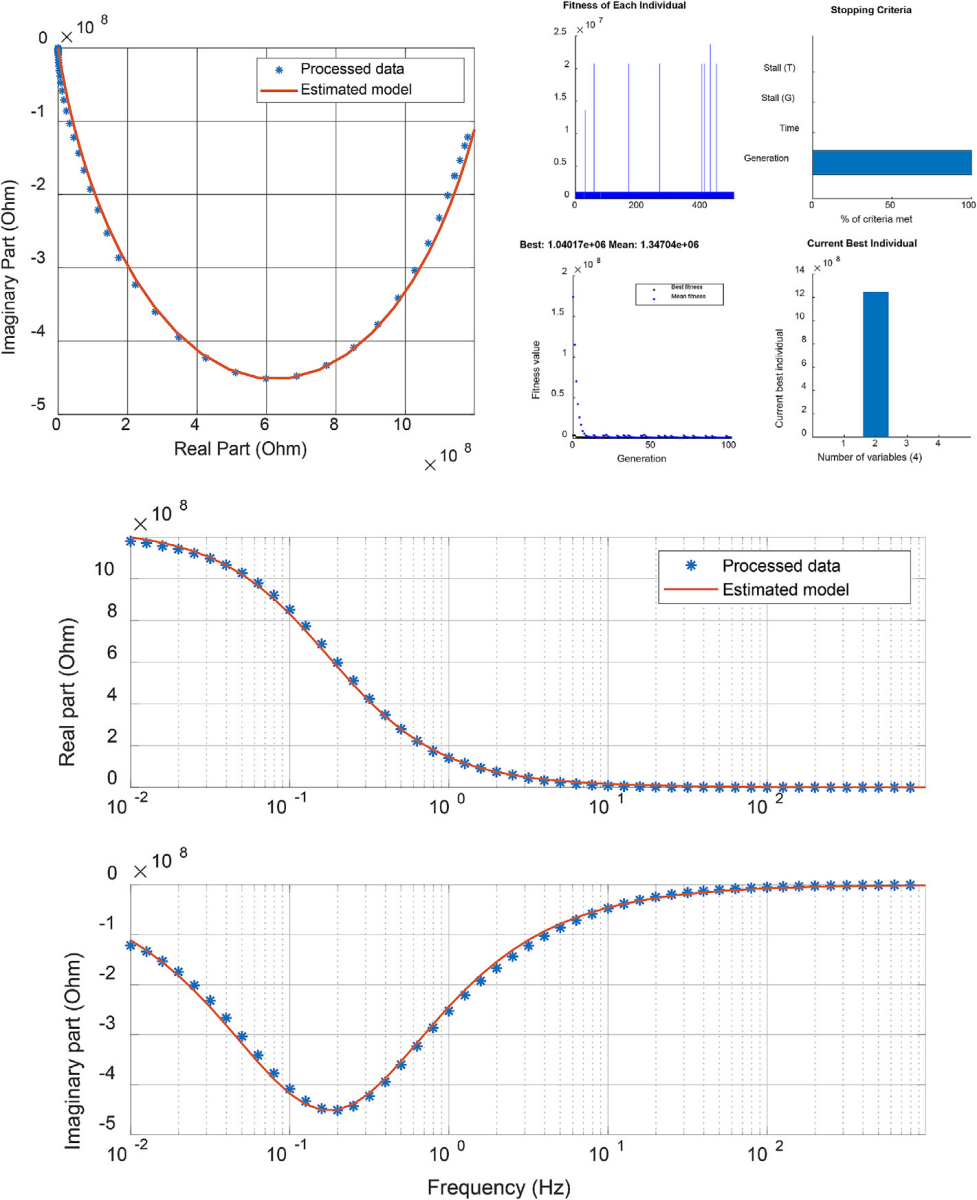
Identification result for the real and imaginary parts of model (21), the equivalent polar plot of model and data for the fat sample (top left), and its GA optimization solver view (top right). The identified model here is $R=48.1727,K=1.2466\cdot 10^{9},p=0.1771,.\beta =0.7987$.

The next step is to validate the hypothesis that the dynamic response varies with fat volumes. In order to perform identification and fit the model (21) to the fat samples, the Normalized Root Mean Square Error (NRMSE) is calculated from each optimal parameter vector generated after each GA iteration. For at least a NRMSE<0.1, the parameters set, that have the highest incidence and that fulfill the condition, were considered for the model initialization in the online procedure for the same patient. Giving a fitting percentage equal to fit=100*(1-NRMSE), the optimization result for one tissue sample is depicted in Fig. 17 (top left and bottom) along with the corresponding best fit for model identification response. An illustrative example of such GA identification result is given in Fig. 17 (top right). The results of all samples are summarized in Table 4. The obtained results indicate a consistent progression of the tissue properties as a function of sample volume.

**Table 4:** Identification results of the 10 fat tissue samples with the model (21). * denotes values times e + 9. ** denotes values times e + 7.

Sample	*R*	K*	*P*	β	τ = ${\left(K/P\right)}^{\beta }$**	$\tau ={\left(1/P\right)}^{\beta }$	τ=(1/P)
1/10	48.0619	0.9944	0.2238	0.7900	4.1849	3.2630	4.4683
2/10	48.0937	1.3002	0.1821	0.7900	6.0873	3.8402	5.4915
3/10	48.0479	1.3002	0.2005	0.8398	17.3792	3.8555	4.9875
4/10	48.0463	1.0996	0.2202	0.7954	5.1775	3.3321	4.5413
5/10	48.0958	1.4545	0.1771	0.8317	17.6151	4.2195	5.6465
6/10	48.0242	1.2805	0.1771	0.8140	10.6012	4.0921	5.6465
7/10	48.1727	1.2466	0.1771	0.7987	7.3317	3.9852	5.6465
8/10	48.0436	1.1063	0.2183	0.7090	0.7598	2.9418	4.5809
9/10	59.8018	1.4086	0.1771	0.8178	12.4935	4.1191	5.6465
10/10	48.0520	1.2805	0.1821	0.7954	6.7977	3.8757	5.4915

### Dynamic response of PK-PD patient model to single bolus input

We use a simplified version for one input (Propofol drug) and one output (BIS values) from the complex open-source patient simulator described in [74]. The Matlab program for this particular study is given in the Supplementary Material file.

**Fig. 18:**
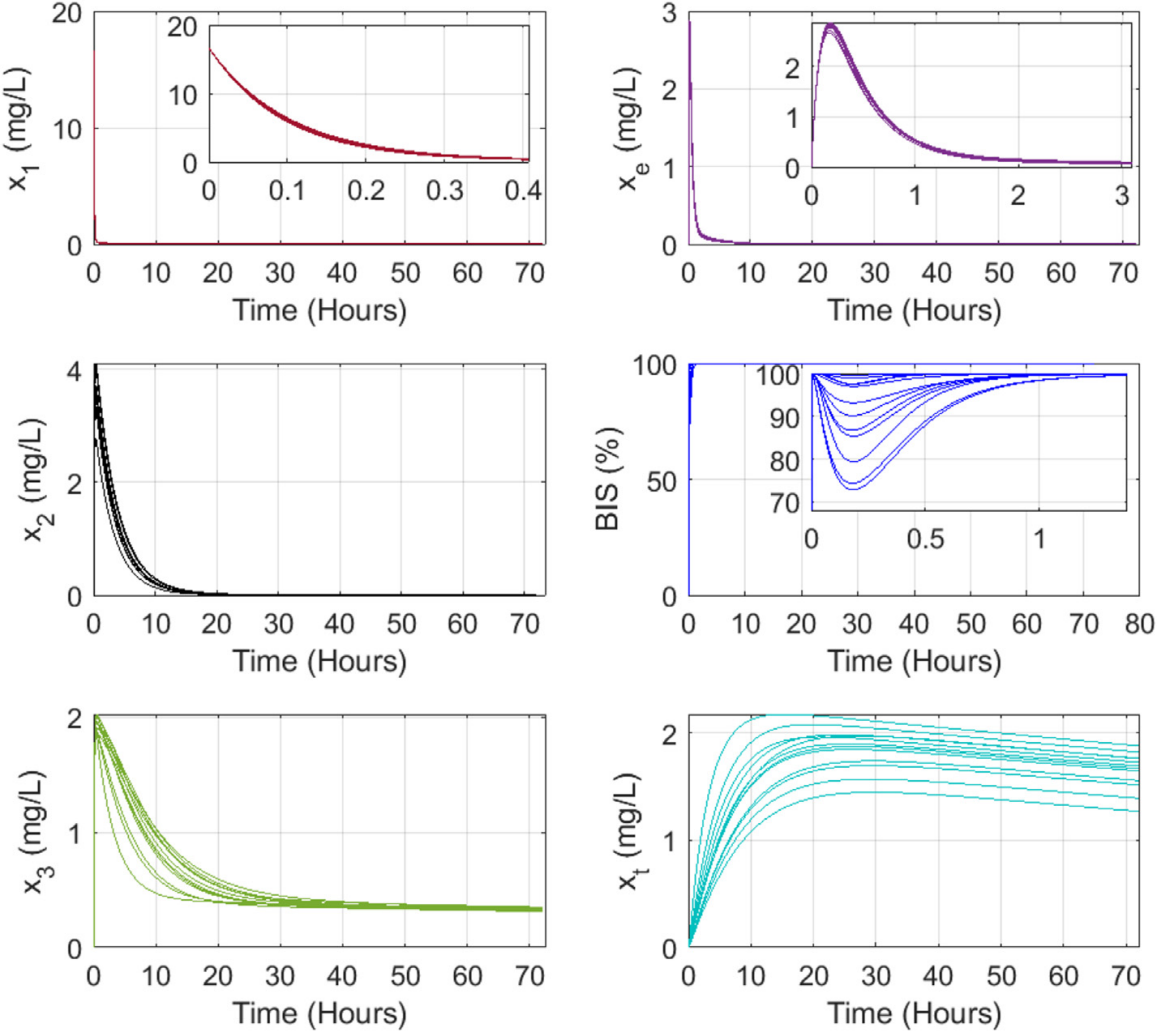
Open loop simulation for 12 patients dataset from Table B.7: The first column shows the evolution of drug concentration in the first three compartments: blood, muscle, and fat. The second column shows the same evolution in the effect site concentration and the fat trap. The last plot represents the BIS as a function of time during 72 h of simulation.

**Fig. 19:**
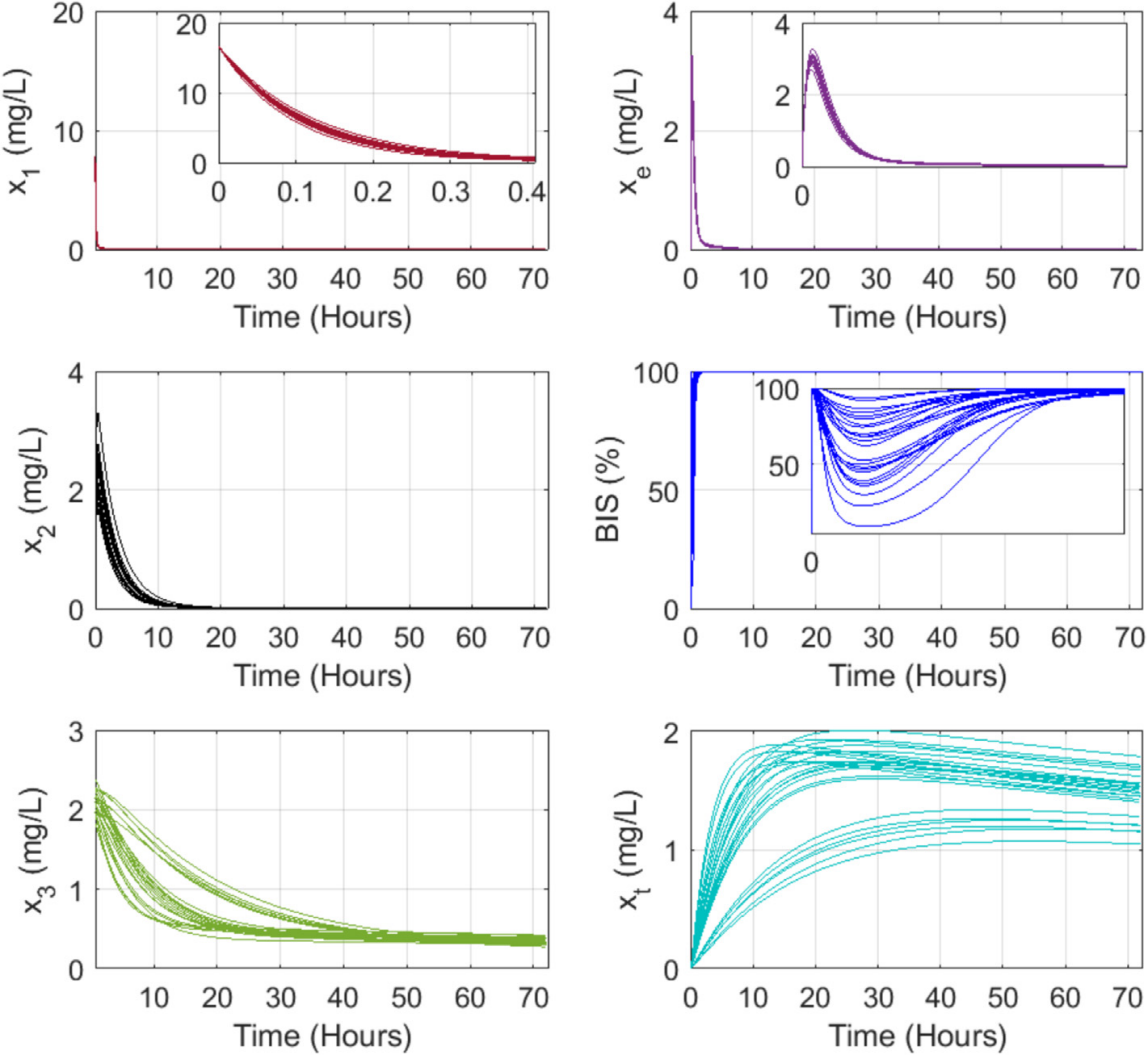
Open loop simulation for 24 patients dataset from Table B.8: The first column shows the evolution of drug concentration in the first three compartments: blood, muscle, and fat. The second column shows the same evolution in the effect site concentration and the fat trap. The last plot represents the BIS as a function of time during 72 h of simulation.

**Fig. 20:**
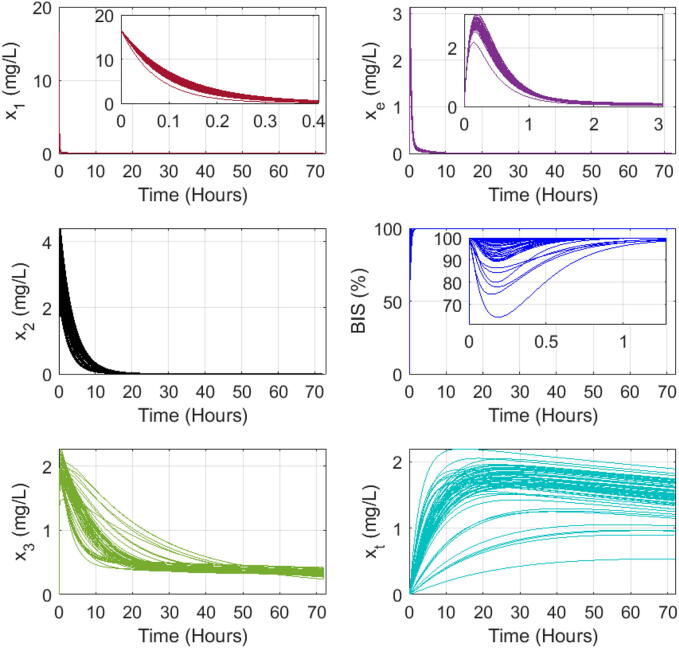
Open loop simulation for 70 patients dataset from Table B.9: The first column shows the evolution of drug concentration in the first three compartments: blood, muscle, and fat. The second column shows the same evolution in the effect site concentration and the fat trap. The last plot represents the BIS as a function of time during 72 h of simulation.

Here we investigate the presence of the long tails expected in drug trapping volume and its effect on the other drug concentrations and output model values. The simulation assumes a bolus input infusion of 3.33 mg/s Propofol dose for 5 s, administered intravenously in the blood compartment. The open loop simulation runs for 72 h, with a sampling period of 1 s. The model equations from (2), (3), (4), (13) use coefficient values from the three databases in Table B.7, Table B.8, and Table B.9, respectively. The interpatient variability is visible in the variations of the responses as a function of each patient’s characteristics, notably the sensitivity to the drug effect captured by nonlinear gain function in (20). Fig. 18, Fig. 19, Fig. 20 show the evolution of the concentrations in the three compartments (blood, muscle, and fat), the effect site concentration with the time constants of drug absorption and clearance correspond to the clinical onset values for each patient from their respective tables, the trapped drug concentration, and the level of anesthesia BIS in patients.

**Fig. 21:**
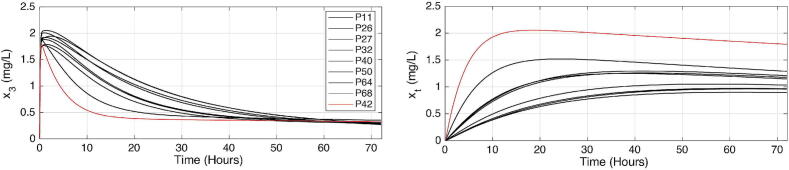
Comparison of the fat trap compartment concentration ($x_{t}$) and the fat compartment concentration ($x_{3}$) clearance rate of patients from Table B.9 that have high BMI (over 30) and a low BMP (P42 with 19.1) in the 70 patient database.

Indeed, the numerical simulations confirm that the concentration of trapped drug clears at a significantly slower rate than the effect site concentration. In the fat compartment Fig. 21, patients with a higher BMI tend to absorb more drugs and retain them longer than individuals with lower BMIs. This result explains why overweight and obese patients need increased drug dosage to achieve the desired level of hypnosis during surgery, but on longer term they tend to be overdosed as part of trapped drug recirculates latently through the liver in the body. Consequently, they often have longer recovery times in intensive care units and experience extended post-surgery side effects.

### Anesthesia maintenance by continuous drug infusion

We simulate here various closed-loop control of anesthesia scenarios to investigate the effect of the additional fat trap volume compartment on the total drug usage and BIS values. In long-term anesthesia, the objective for control is to maintain the BIS values within a safe interval between 40 and 60, while in presence of few to no disturbance stimuli. In our numerical simulation, we will consider a disturbance free environment, as to clearly observe the effects of patient model characteristics in the closed loop performance.

The total time of simulation is for 20 min, as this corresponds to a reasonable prediction of events during intensive care under anesthesiologist supervision. The computer control uses a fast sampling time of 5 s. The prediction horizon used in the optimizer block of EPSAC has been tuned based on the user-friendly hands on tuning rules, i.e. about three times the transient time, as to cover the combined blood and effect site compartment time constants [70] as main dynamic transients of the overall system.

**Fig. 22:**
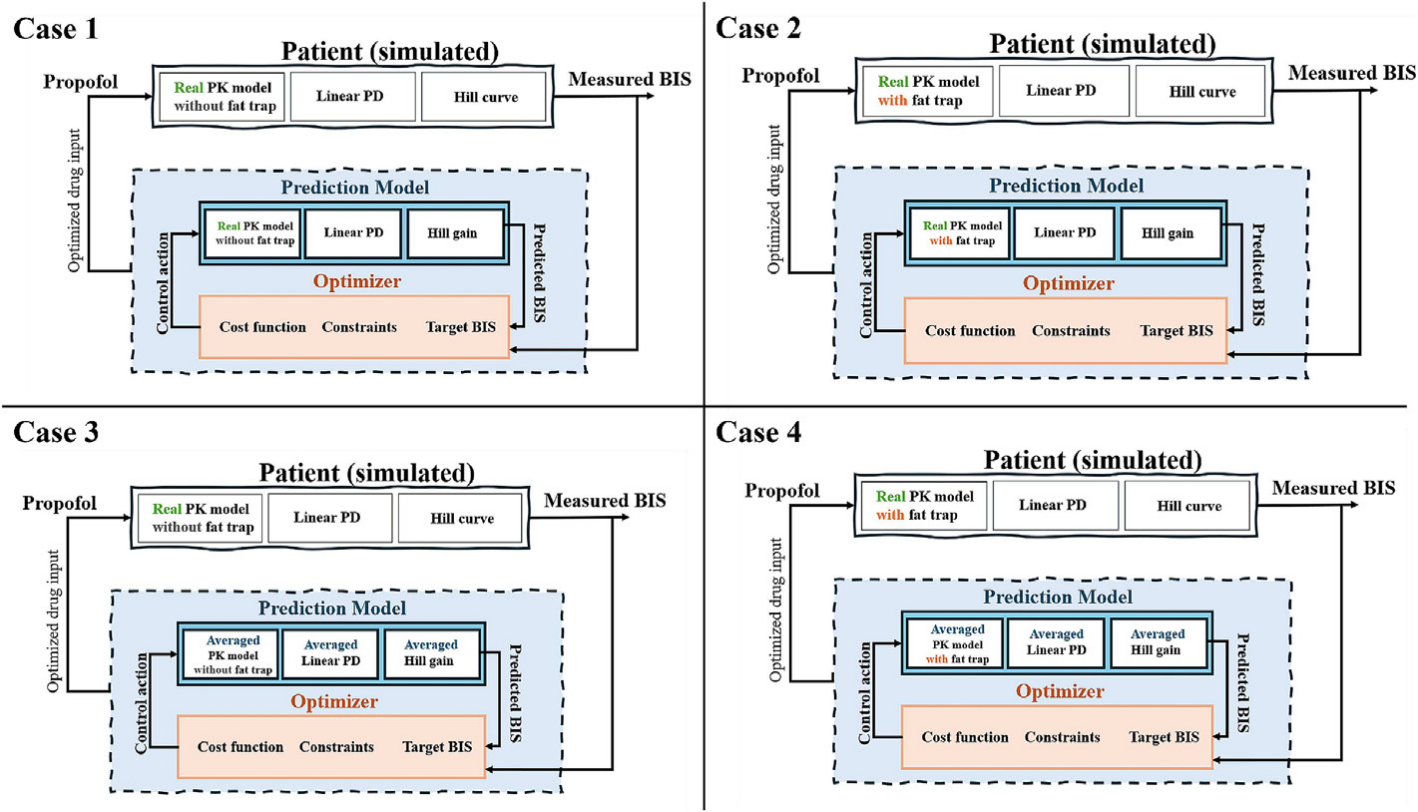
Overview of the 4 situations we test in numerical simulation for comparison analysis.

The simulation scenarios are summarized in Fig. 22. Notice the combinations of real patient model and averaged patient model, as well as combinations of with and without the augmented fat trap volume compartment.

**Fig. 23:**
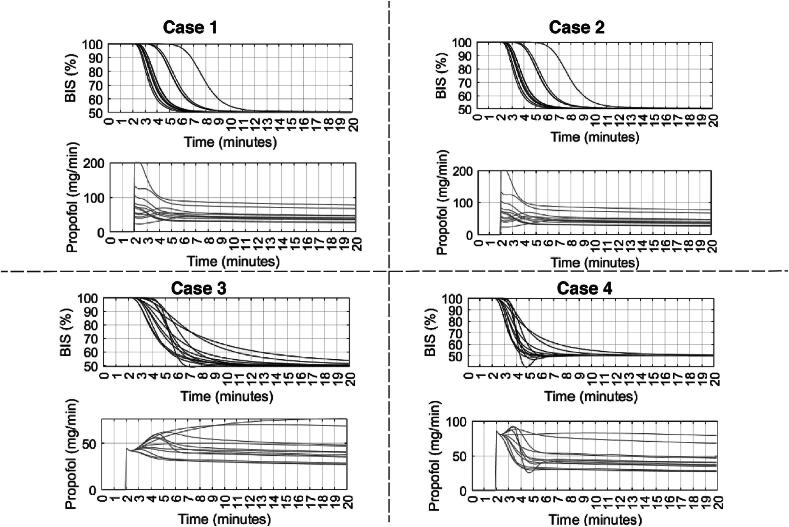
Numerical result for 12 patients database in closed loop simulation: output values (BIS) and input drug usage (Propofol).

**Fig. 24:**
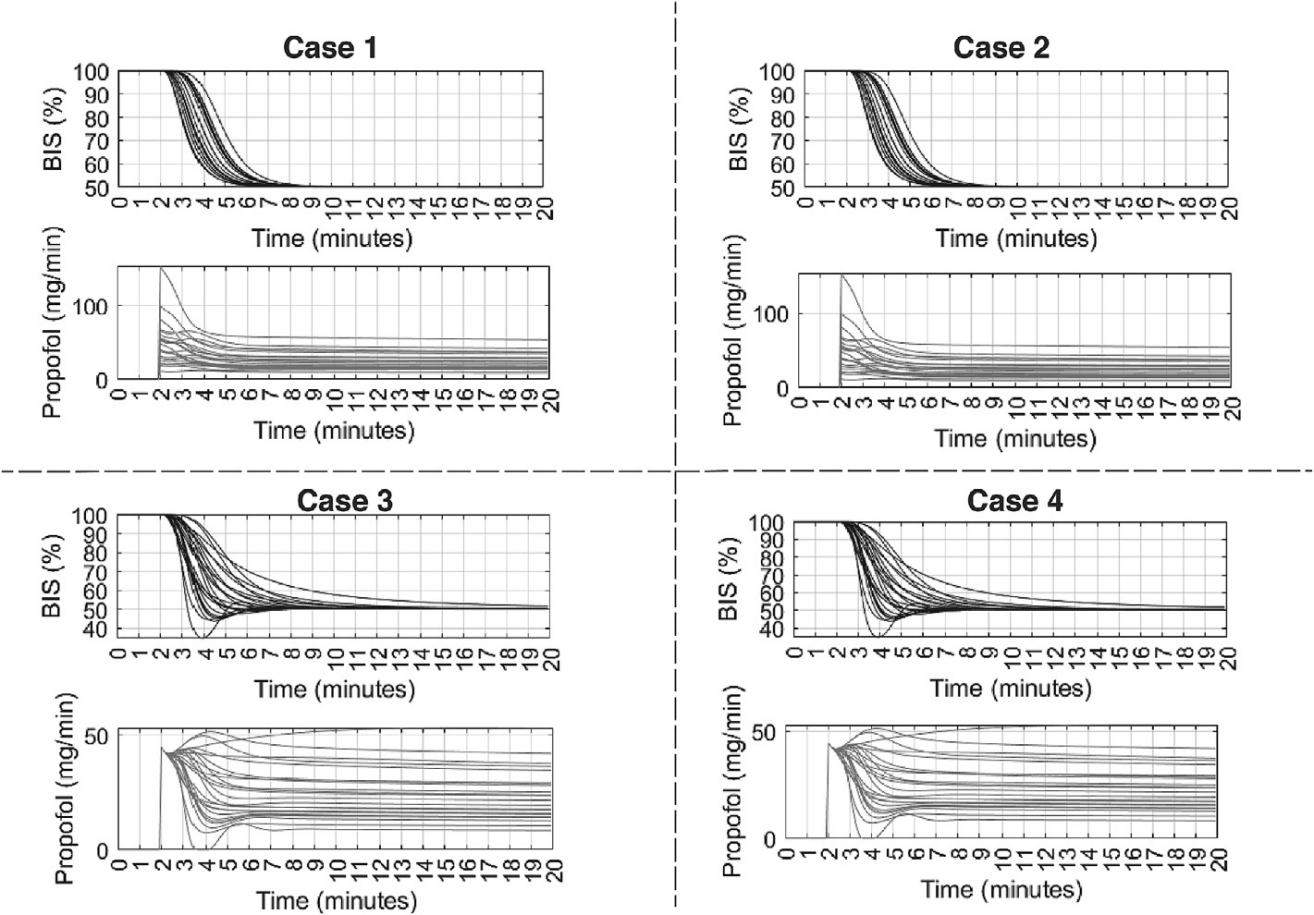
Numerical result for 24 patients database in closed loop simulation: output values (BIS) and input drug usage (Propofol).

**Fig. 25:**
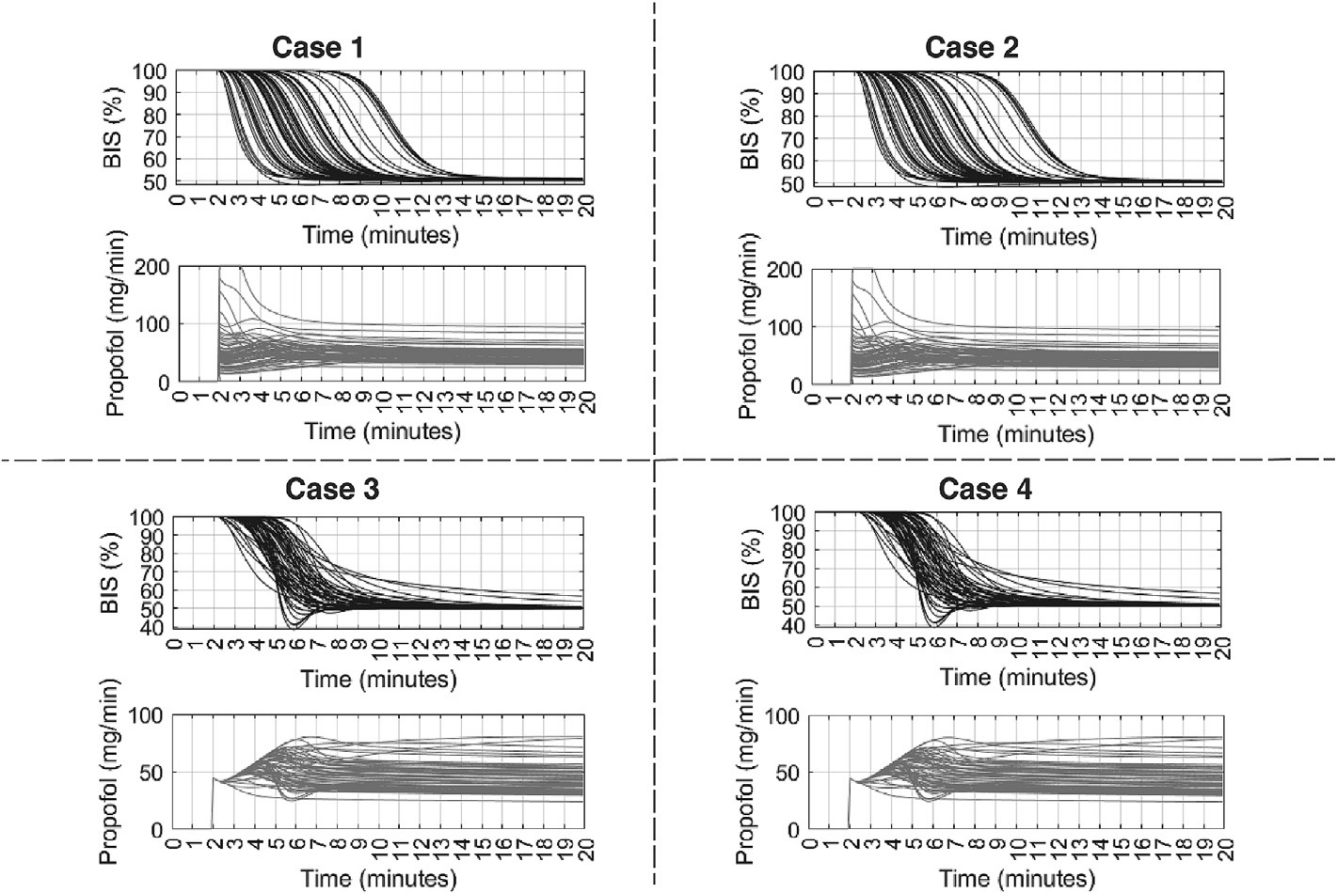
Numerical result for 70 patients database in closed loop simulation: output values (BIS) and input drug usage (Propofol).

Fig. 23, Fig. 24, Fig. 25 depict the BIS values (output) and Propofol infusion (input) for each case in the 12, 24, and 70 databases respectively. The results show that the augmented model with trap fat volume does not influence the control performance. This can be explained by the fact that the controller uses prediction horizons based on the main time constant of the system, namely the patient in our case, which corresponds to the effect site compartment related to the fast compartment (blood). In contrast, longer time constants from the fat and trap fat compartments are intrinsically compensated through the closed-loop feedback error. Additionally, when we use the classical model without any trap fat volume in either models, we observe a slight difference in the controller output, respectively, a difference in drug management profiles.

**Fig. 26:**
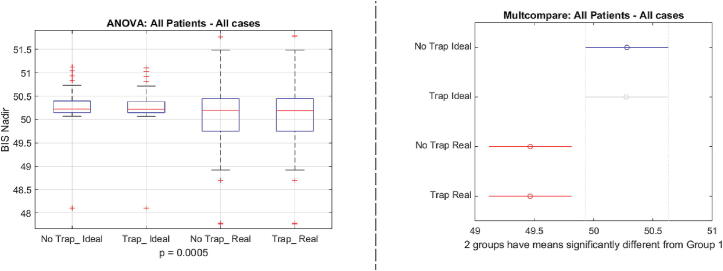
One way analysis of variance and comparative analysis of variance for the BIS nadir values in the 4 cases.

**Fig. 27:**
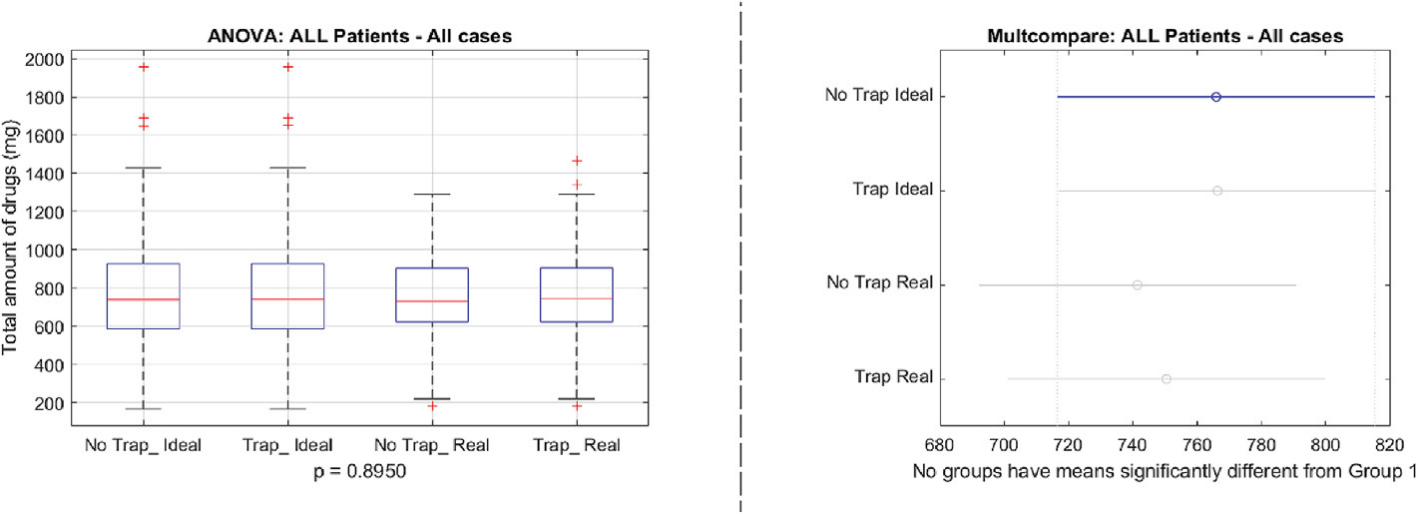
One way analysis of variance and comparative analysis of variance for the total drug usage values in the 4 cases.

To examine the difference between the 4 scenarios, we use the performance metrics in (25) and in (26). Fig. 26 indicates a significant difference between the ideal case and the real case situations p=0.0005, but no significant difference in the usage of the augmented fat trap volume model from (13). When examining the total drug usage per case, we obtain the results depicted by Fig. 27, where no significant difference has been observed p=0.8950.

**Fig. 28:**
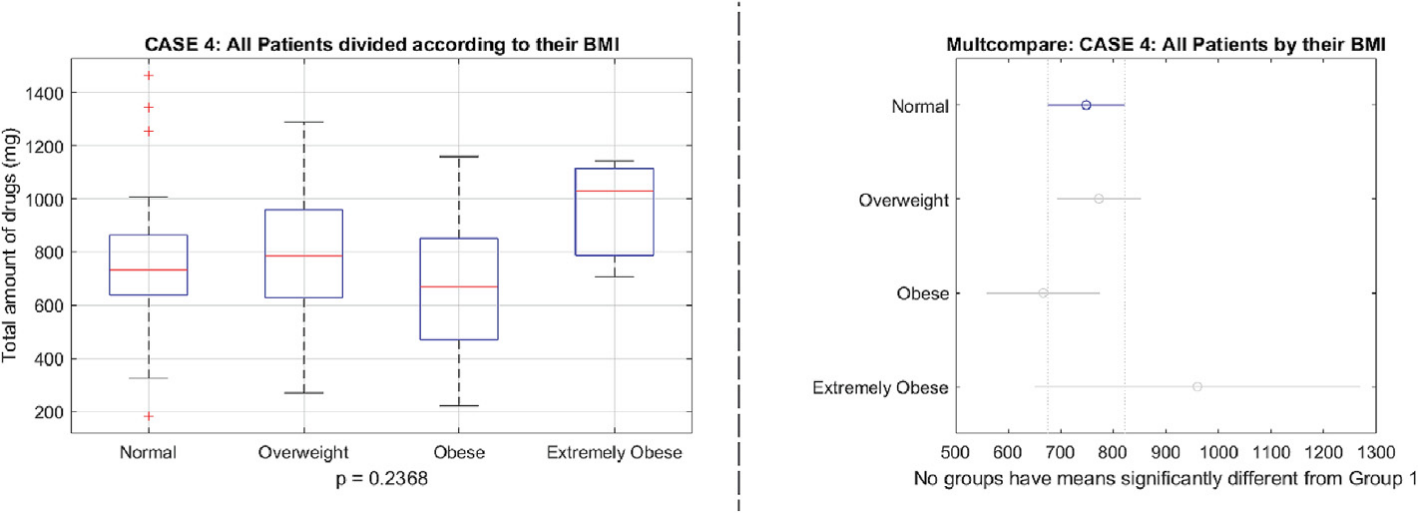
One way analysis of variance and comparative analysis of variance for the total drug usage values as a function of BMI classes of patients.

A comparison of total amount of drug usage distributed as a function of BMI is given in Fig. 28. There are visible differences in the median values, but no statistically significant difference p=0.2368. This is possibly due to relatively small number of patients in each category and one may speculate that the differences become more pronounced as the number of data samples increases.

**Fig. 29:**
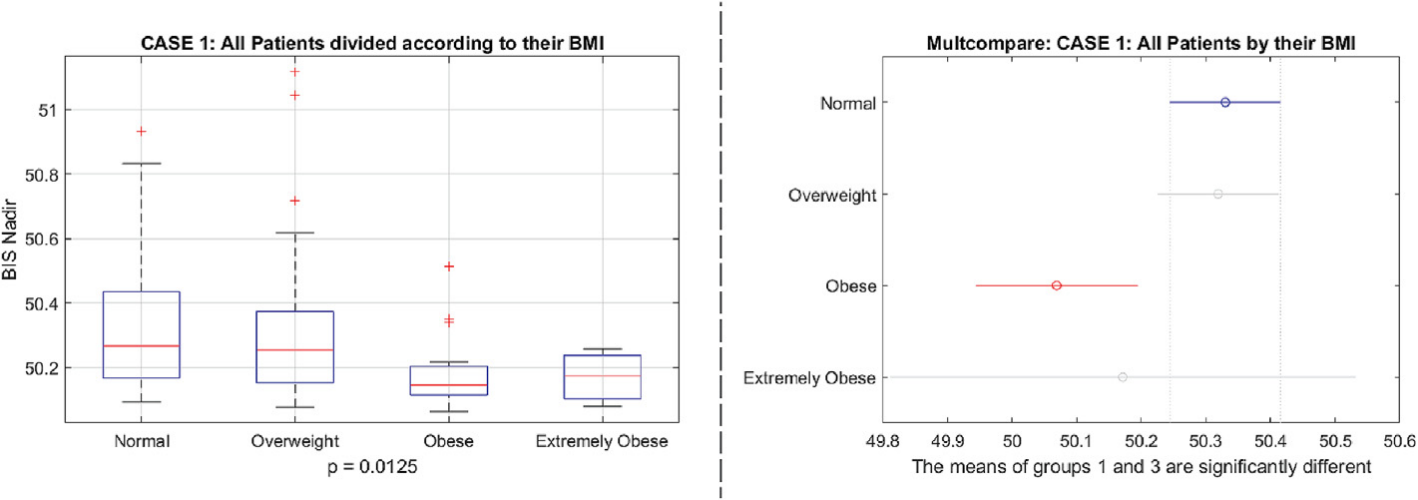
Case 1 results: one way analysis of variance and comparative analysis of variance for the BIS nadir values as a function of BMI classes of patients.

**Fig. 30:**
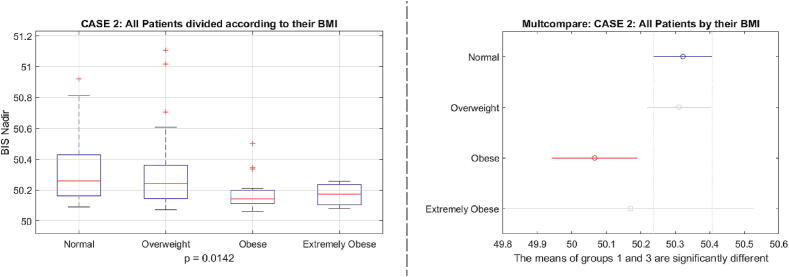
Case 2 results: one way analysis of variance and comparative analysis of variance for the BIS nadir values as a function of BMI classes of patients.

When we examine the closed loop performance in terms of BIS nadir value as a function of BMI, we have significant difference p=0.0125 between classes in case 1 depicted in Fig. 29, and significant differences p=0.0142 in case 2 depicted in Fig. 30.

**Fig. 31:**
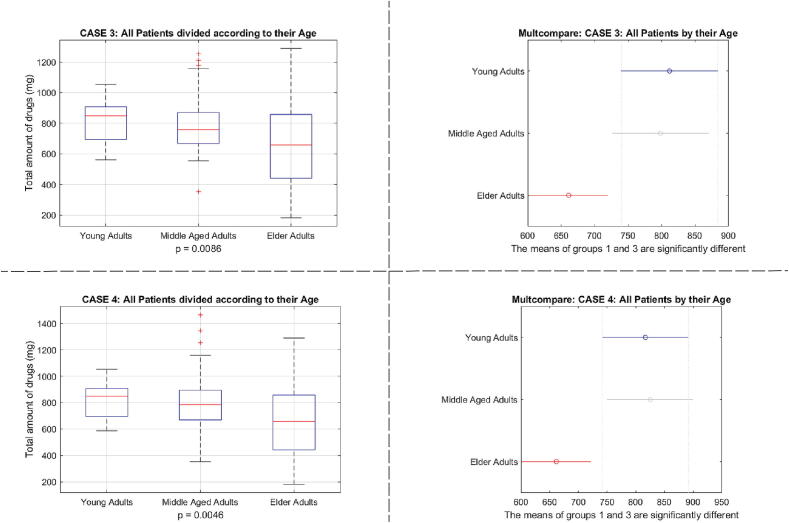
One way analysis of variance and comparative analysis of variance for total drug usage as a function of Age classes of patients.

**Fig. 32:**
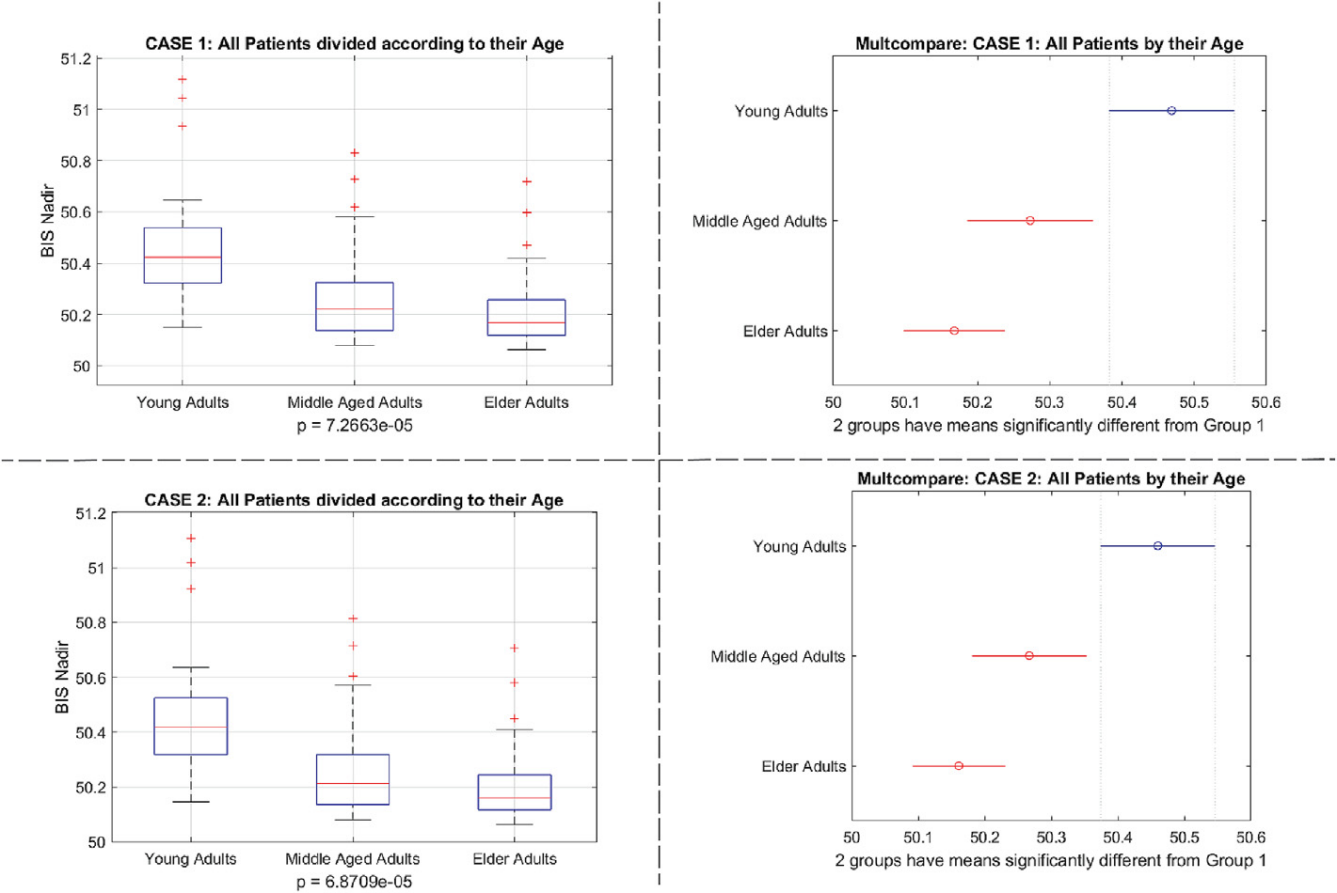
One way analysis of variance and comparative analysis of variance for the BIS nadir values as a function of Age classes of patients.

As expected, a difference was obtained between young and elderly groups of patients and in BIS nadir values. The results are given in Fig. 31, Fig. 32, respectively. As a function of Age, the total drug usage was significantly different for case 3 and case 4 with p≪0.001, but not between cases for same age groups. This suggests the difference in drug usage comes mainly from the modelling errors between real process and predicted model rather than the augmented fat trap volume.

Observing Fig. 32, we see that as a function of Age, the BIS nadir values were significantly different for case 1 and case 2 with p≪0.001. As there were not modelling errors (ideal cases) in these cases, this suggests the difference in output effect performance comes mainly from whether or not the augmented fat trap volume was present.

More details are to be found in the Supplementary Material file.

## Discussion

The results of the fractional order impedance model from the examined fat samples show that the resistance (R) is relatively low, suggesting that the fat sample exhibits good conductivity. The strong polarization (K) indicates that fat tissue exhibits a significant polarization effect and has capacitive behavior. This high capacitance is indicative of the ability of the fat sample to store electrical energy. In biological contexts, this could be related to the structural composition of the fat, including aspects like cell membrane properties and the presence of various types of lipids which can affect dielectric properties. Generalization implies it affects the time constant of binding molecules in heterogeneous substrates of tissue cells [75]. The decrease in the resonant frequency (P) with the volume of the fat sample tested indicates the time constant increases as the volume increases. Extrapolation to the fat volume in patients provides a direct linear proportional agreement with the experimental values and corresponds to the theoretical assumptions of this work.

The Cole–Cole exponent (β) is close to 1, which means the tissue has a distribution of relaxation times that is fairly narrow, indicating significant non-ideal (non-Debye) behavior [76]. This suggests that the electrical response of the fat sample is complex and does not conform to simple capacitive models, something that we already expected and now confirmed. In the context of the Cole–Cole model, the term *anomalous diffusion* refers to a deviation from the simple Debye behavior, which is characterized by a single relaxation time. Anomalous diffusion can be related to the complexity of the medium through which molecules or ions move, leading to a non-uniform distribution of relaxation times [77]. Considering the given parameter β=0.838, we infer that the behavior is not perfectly Debye, which implies that the diffusion might not be completely normal and hints at some degree of heterogeneity in the fat tissue. Indeed, as explained in the background section of this paper, the fat cells evolve in time and change their permeability properties, while affecting aggregation (irregular clusters of molecules).

The fractional order in the Cole–Cole model and the long tails in dynamical response in the concentrations of fat volumes in our PK models indicates existence of memory and suggest that other forms of PK models may be applied. For instance, PK models with explicit memory terms [78] and fractional order differential equations for anomalous diffusion patterns and heterogeneous structures [79].

Recall Fig. 21, where the left-plot shows the evolution of drug concentrations over time for the fat compartment $x_{3}$ as a function of time. The red line represents a patient with a normal-to-low BMI=19.1 while the black lines represent obese and extremely obese patients (BMI>30). From this plot, we observe that the uptake and clearance rates of the drug in the fat compartment are faster for the very lean patient compared to the obese and extremely obese patients. This suggests that lean patients absorb and release the drug faster from the fat compartment, leading to larger peak values for shorter time intervals. The right plot illustrates the augmented fat trap compartment $x_{t}$. We observe that the lean patient absorbs the drugs faster, leading to a higher concentration in this compartment, which aligns with the *sponge theory*: a dry sponge absorbs faster and more water than an already wet one, previously discussed in [78]. The complex properties of the highly heterogeneous fat tissue in patients with BMI>25 will clearly affect the uptake and clearance rates in the fat volumes and residence times of trapped drug molecules may be associated with self-reinforcement tissue adaptation, yet another strong property of fractional master equations and memory effects [80].

**Fig. 33:**
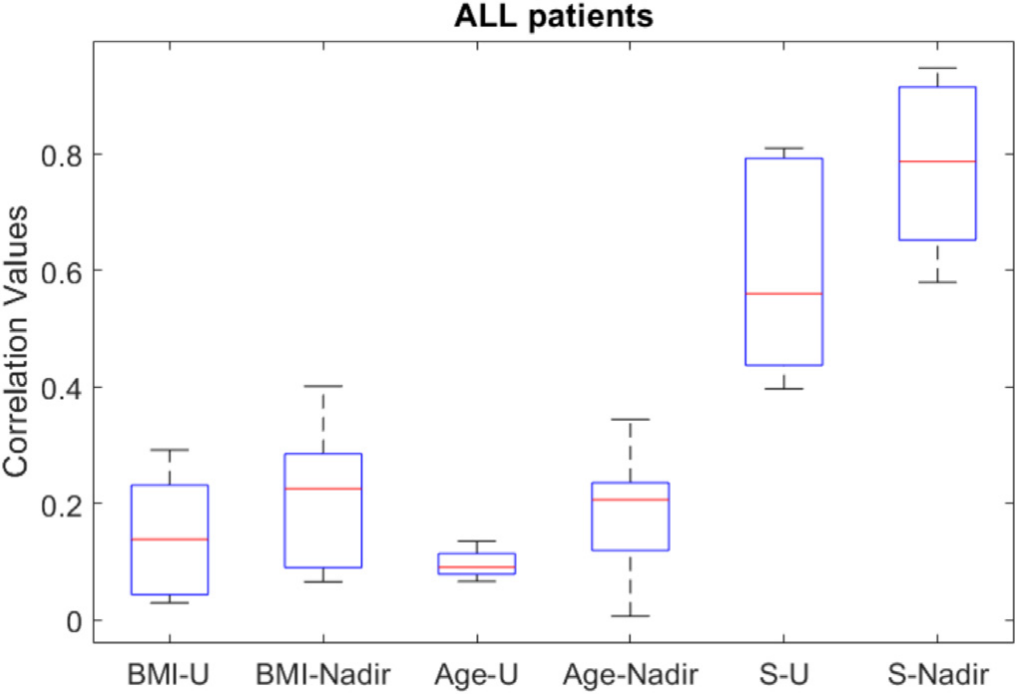
Correlation values between various indicators, for all patients. See text for details.

These molecular properties play a crucial role in determining the sensitivity or the resistivity of the patient to the dose–effect of the administered drug and explains largely the underlying mechanism of patient intervariability, as well as intravariability. In our PD model we have the slope of the nonlinear Hill curve characterising the dose–effect in each patient, to which we extract the linear gain around the maintenance values of the output of general anesthesia, BIS = 50%. Very strong correlations are observed in Fig. 33 between this gain *S* (representing the sensitivity of the patient to the dose–effect) with the closed loop performance BIS nadir and the total drug usage for each patient.

As previously defined in Eqs. (24), (25), (26), Fig. 33 presents the correlation values between patient characteristics (BMI, Age, and sensitivity S) and two clinical indicators: total drug usage (U) and BIS nadir values. The x-axis displays six different correlation pairs (BMI-U, BMI-Nadir, Age-U, Age-Nadir, S-U, S-Nadir), while the y-axis shows the corresponding correlation coefficients. This figure provides consistently lower correlation values in total drug usage than in output variable BIS nadir, suggesting the controller has intrinsic robustness in manipulated variable variability. The BMI seemed to influence more than Age in both total drug usage as well as BIS nadir values. As expected, Age plays a significant role in determining overall performance indicators as it has the main influence on the coefficients of the PK model uptake and clearance rates.

From all the closed loop simulations and all statistical analysis reported in previous section we conclude that differences in total drug usage are rather associated with modelling errors rather than with the existence of drug trapping model equation in the simulation test. There were no significant differences observed between cases as a function of their BMI category, which suggests the closed loop compensates rather well among modelling errors from the existence or absence of fat trap volume. This is explained by the iterative nature of the predictive control, which reiterates the optimal values of drug input every sample for the entire length of the prediction horizon. Since the dynamic effects of the trapping of molecules is a slow process, far beyond the time constants of the dominant compartments (blood and effect-site), these effects are compensated in closed loop control. Still, this is due in part to the disturbance free environment of testing in this work. Long-term (>6 h) anesthesia in surgery conditions will clearly affect the outcomes differently in performance indicators and correlations, but this requires specific clinical data availability for analysis.

Several limitations are present in this study. We start by mentioning that the selected fat samples under observation in our experimental setup have been chosen to be rather homogeneous in their structure and with relative close density between intermediate sample volumes. It should be interesting to examine fat samples in a larger volume ensemble consisting of variations in both density and structural assembly. This will give insight into possible effects on the time constant and extrapolate to volumes and classes of BMI in patients. Another limitation is the fact that the position of the patient has not been taken into account; often, patients are laid in supine position or prone position (COVID-19 patients) and this affects the fluid shifts and accumulations in the body and in afferent tissue volumes. Finally, hemodynamic stabilisation with fluid volume management affects the properties of the adipose and fat tissue, hence expected to alter time constants and diffusion mechanisms at molecular level. Our simulator proposed in [74] is currently undergoing revision to include these important characteristics in our endeavour to mimic closely general anesthesia conditions and provide a close-by digital twin of this complex process.

## Conclusions

In this paper, we introduced a theoretical background for BMI dependent time constant dynamics variability in pharmacokinetic modelling. We validated our theory at laboratory scale by in vitro measurement of fat samples of various volumes. The numerical simulations for single bolus infusion depict the typical long tails of slow dynamical clearance of drug from tissues with slow acting molecular binding and suggest low to sub-diffusion patterns with memory effects. Closed loop numerical simulations for continuous drug infusion management illustrate the potential of closed loop control to eliminate these effects from the realistic time interval of general anesthesia management. The study emphasizes the importance of minimizing modelling errors between the simulated actual patient (real) and the patient PK-PD model used for optimization of drug profiles. The discussion section provides a link between our findings and interdisciplinary domains of application of this work in mathematics, medicine and engineering.

The augmented PK model provides a more physiologically grounded representation of drug retention by accounting for BMI-dependent nonlinearities in distribution and clearance. One advantage of this approach is that it enhances control performance by reducing overall drug usage and improving anesthetic depth tracking, particularly under long-term infusion protocols. Another advantage is that it can be used for clinical simulation, helping to predict drug dynamics across a diverse patient population. Simulation results based on clinical data from over 100 patients confirmed delayed drug clearance in high-BMI individuals and revealed significant differences in BIS nadir values and total drug input across BMI and age groups. These findings support the model’s relevance in comorbid conditions and its suitability for optimizing infusion profiles. Compared to classical PK models, the augmented model captures BMI-dependent nonlinearities in drug retention and clearance. This allows for more accurate long-term predictions of drug behavior and improves control performance during anesthesia management. Existing models often overlook the impact of comorbidities such as obesity and do not account for the phenomenon of drug trapping in adipose tissue, which can significantly alter pharmacokinetics during prolonged infusions. By explicitly modeling this effect through a BMI-dependent trap compartment, the proposed scheme offers improved physiological realism and greater reliability in managing anesthesia in patients with elevated BMI. The main disadvantage of the model is its increased structural complexity, which introduces additional parameters that must be calibrated to patient-specific data to preserve accuracy and enable meaningful real-time application.

Further work is focused on resolving the current limitations of the study. In particular, the model will be extended to simulate longer anesthesia durations and include additional drugs such as analgesics and neuromuscular blockers. Integration with dynamic hemodynamic models will be considered to account for patient-specific cardiovascular responses. Comparative analysis with fractional-order compartmental models, known for capturing memory and anomalous diffusion behaviors in drug kinetics, will also be explored to improve model fidelity. The lack of a sensor to directly measure circulating drug concentration remains a constraint for real-time validation. A key direction will be the development of a digital twin framework that combines this model with real-time physiological monitoring (e.g., heart rate, blood pressure, BIS index) to enable adaptive and personalized drug delivery under closed-loop control.

## Compliance with ethics requirements

Ethical committee approval for the patients databases with real data.

For the 24 patient database, the clinical related information can be found on ClinicalTrials.gov/NCT00735631, and was compliant with the regulatory framework stated in the European Regulation (EU) 2017/745, and approved by the Ethics Committee of Ghent University Hospital, Belgium.

For the database of 70 patients, the clinical investigation involving human subjects was compliant with the regulatory framework stated in the European Regulation (EU) 2017/745. This academic clinical investigation was approved by the Ethics Committee of Ghent University Hospital and the Federal Agency for Medicines and Health Products of Belgium FAGG (EC/BC-08020, FAGG/80M0840, EudraCT: CIV-BE-20-07-0342442020, clinicaltrials.gov: NCT04986163, principal investigator: Martine Neckebroek).

## Credit author statement

**Amani Ynineb:** Software, investigation, writing, validation, methodology. **Erhan Yumuk:** Software, methodology Dana Copot: Software, methodology. **Ghada Ben Othman:** Software, methodology, review, editing. **Hamed Farbakhsh:** review and editing. **Isabela Birs:** review and editing. **Robin De Keyser:** Software, review. **Samir Ladaci:** methodology. **Cristina Muresan:** Methodology. **Martine Neckebroek:** Methodology, review, writing, investigation. **Clara Ionescu:** conceptualization, supervision, software, review and editing.

## Declaration of Competing Interest

The authors declare that they have no known competing financial interests or personal relationships that could have appeared to influence the work reported in this paper.

## Figures and Tables

**Fig. A.34:**
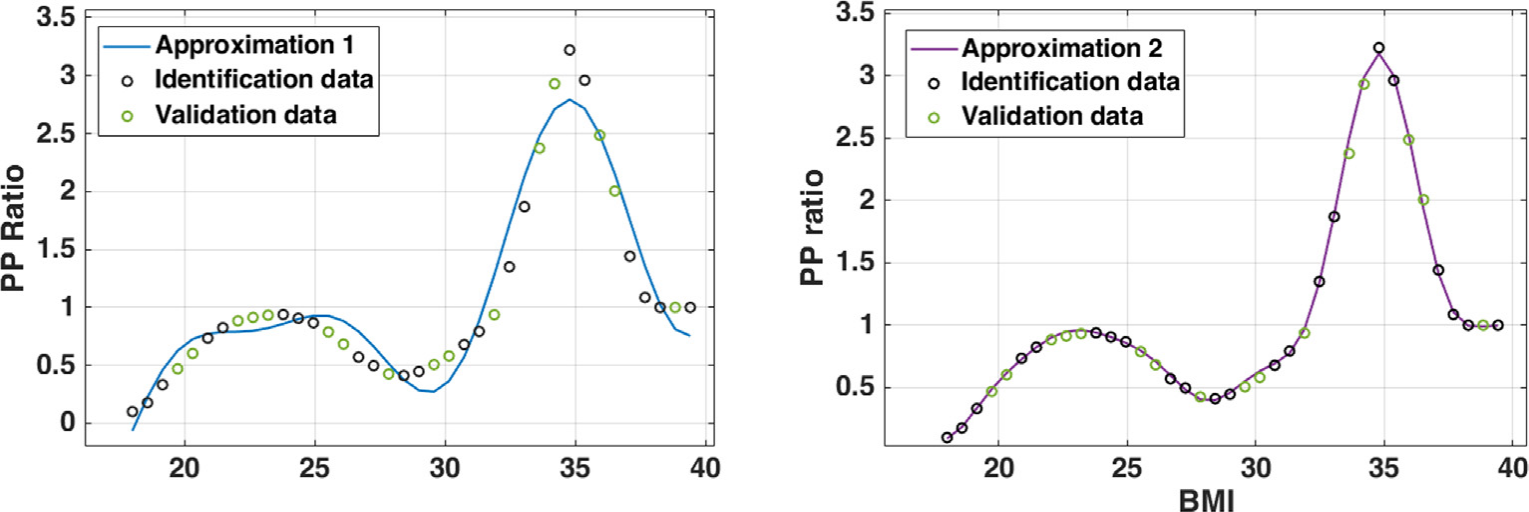
Comparison between two approximations from Table A.6 using identification and validation data Sets for the relationship between the PP Ratio and BMI.

**Table A.5:** Performance measures of the approximations for goodness of identification (70% of data), goodness of validation (30% of data) and global fit (100% of data).

	Goodness of Identification			Goodness of validation		Global Fit	
Approximation	SSE	R-square	RMSE	SSE	RMSE	NRMSE	Fit
Approx1: 3 sine	0.8468	0.9356	0.2552	0.4823	0.1736	0.0600	94.0005
Approx2: 6 sine	0.0070	0.9995	0.0418	0.0357	0.0472	0.0108	98.9246

**Table A.6:** Model coefficients.

Approximation 1: 3 sinusoids m = 3					
$a_{1}$	27.4472	$a_{2}$	0.8002	$a_{3}$	0.4815
$b_{1}$	0.0033	$b_{2}$	0.5390	$b_{3}$	0.7595
$c_{1}$	−0.0570	$c_{2}$	−4.2180	$c_{3}$	−6.2212
					
Approximation 1: 6 sinusoids m = 6					
$a_{1}$	1.5323	$a_{2}$	0.7569	$a_{3}$	0.8676
$b_{1}$	0.1068	$b_{2}$	0.3100	$b_{3}$	0.6957
$c_{1}$	−1.8903	$c_{2}$	2.4903	$c_{3}$	−2.9134
$a_{4}$	0.1462	$a_{5}$	0.6010	$a_{6}$	0.2260
$b_{4}$	1.4868	$b_{5}$	0.8387	$b_{6}$	1.3274
$c_{4}$	−0.5250	$c_{5}$	2.8708	$c_{6}$	−6.1737

**Table B.7:** Representative 12 Virtual Patient Database with PK Model Biometric Values and PD Model Sensitivity Values from [58].

Index	Gender	Age	Height	Weight	BMI	LBM	$C_{50}$	γ	$E_{0}$	$E_{\max}$
		(years)	(cm)	(kg)	($\mathrm{kg}/m^{2}$)		(μg/mL)			
1	F	40	163	54	20.3	42	6.33	2.24	98.80	94.10
2	F	36	163	50	18.8	40	6.76	4.29	98.60	86.00
3	F	28	164	52	19.3	41	8.44	4.1	91.20	80.70
4	F	50	163	83	31.2	50	6.44	2.18	95.90	102.00
5	M	28	164	60	22.3	49	4.93	2.46	94.70	85.30
6	F	43	163	59	22.2	44	12.10	2.42	90.20	147.00
7	M	37	187	75	21.4	62	8.02	2.10	92.00	104.00
8	F	38	174	80	26.4	54	6.56	4.12	95.50	76.40
9	F	41	170	70	24.2	50	6.15	6.89	89.20	63.80
10	F	37	167	58	20.8	44	13.70	1.65	83.10	151.00
11	M	42	179	78	24.3	61	4.82	1.85	91.80	77.90
12	F	34	172	58	19.6	45	4.95	1.84	96.20	90.80

**Table B.8:** Representative 24 Patient Database (all males) with PK Model Biometric Values and PD Model Sensitivity Values from [74], [42].

Index	Age	Height	Weight	BMI	LBM	$C_{50}$	γ
-	(yrs)	(cm)	(kg)	($\mathrm{kg}/m^{2}$)	-	(μg/ml)	-
1	74	164	88	32.7	60	2.5	3
2	67	161	69	26.6	53	4.6	2
3	75	176	101	32.6	69	5	1.6
4	69	173	97	32.4	67	1.8	2.5
5	45	171	64	21.9	52	6.8	1.78
6	57	182	80	24.2	62	2.7	2.8
7	74	155	55	22.9	44	1.7	3.5
8	71	172	78	26.4	60	7.8	2.9
9	65	176	77	24.9	60	2.9	1.88
10	72	192	73	19.8	62	3.9	3.1
11	69	168	84	29.8	60	2.3	3.1
12	60	190	92	25.5	71	4.8	2.1
13	61	177	81	25.9	62	2.5	3
14	54	173	86	28.1	62	2.5	3
15	71	172	83	28.1	62	4.3	1.9
16	53	186	114	33	77	2.7	1.6
17	72	162	87	33.2	59	4.5	2.9
18	61	182	93	28.1	69	2.7	1.78
19	70	167	77	27.6	58	6.8	3.1
20	69	168	82	29.1	60	9.8	1.6
21	69	158	81	32.4	55	3.2	2.1
22	60	165	85	31.2	60	5.1	2.51
23	70	173	69	23.1	56	3.67	3.1
24	56	186	99	28.6	73	5.8	2.3

**Table B.9:** Representative 70 Patient Database with PK Model Biometric Values and PD Model Sensitivity Values (1/2)

Index	Gender	Age	Height	Weight	BMI	LBM	$C_{50}$	γ	$E_{0}$
		(years)	(cm)	(kg)	($\mathrm{kg}/m^{2}$)	-	(μg/ml)		
1	F	31	163	49	18.4	39	7.7	5.8	97.7
2	F	44	168	57	20.2	44	5.7	3.3	96.5
3	F	42	168	70	24.8	49	6.4	3	97.6
4	F	71	158	69	27.6	46	9.5	7.1	86.3
5	F	38	182	87.6	26.4	59	6.3	2.7	94.2
6	F	41	175	135	44.1	56	4.7	1.4	97.3
7	F	68	172	67	22.6	49	5.8	4.3	97
8	M	59	183	76	22.7	62	4.3	1.6	80.9
9	M	50	186	96	27.7	72	5.6	4.8	92.2
10	M	73	181	83	25.3	64	15	2.1	97.6
11	X	22	165	87	32	52	5	3.9	97.3
12	M	29	183	92	27.5	69	7.6	4.1	91.4
13	F	54	160	48	18.8	38	9.3	6.4	94.1
14	X	19	168.7	59	20.7	45	7.9	2.6	94.4
15	X	26	155	61	25.4	42	7.5	5.5	97.7
16	F	54	163	58	21.8	43	6.4	5.9	97.4
17	F	50	174	64	21.1	48	6	1.7	94.1
18	F	30	176	74	23.9	53	5.5	4.8	94.7
19	F	57	164	70	26	48	5.3	9.7	92
20	X	33	162	80	30.5	50	7.8	2.7	97.3
21	F	62	168	88	31.2	54	5.6	3.7	97.7
22	F	48	155	56	23.3	41	7.8	4	97.7
23	M	62	183	85	25.4	66	5.9	3.1	93.9
24	F	36	168	63	22.3	47	8.6	1.5	97.7
25	M	58	179	94	29.3	68	11.3	6.3	95.5
26	F	65	162	87	33.2	50	15	1.1	83.6
27	F	49	167	86	30.8	53	7	2.3	95.6
28	M	46	187	97	27.7	72	10.5	3.7	97.2
29	M	68	176	85	27.4	64	9.7	3.7	91.4
30	F	50	167	70	25.1	49	8.9	4.6	90.5
31	M	54	175	90	29.4	65	8.1	3.9	89.9
32	F	64	164	96	35.7	52	9.5	5	82.9
33	F	46	170	82	28.4	53	8.1	4.1	97.7
34	F	58	157	53.5	21.7	40	7.6	2.6	97.4
35	F	41	167	65	23.3	47	7.5	8.2	92.5

**Table B.10:** Representative 70 Patient Database with PK Model Biometric Values and PD Model Sensitivity Values (2/2).

Index	Gender	Age	Height	Weight	BMI	LBM	$C_{50}$	γ	$E_{0}$
		(years)	(cm)	(kg)	($\mathrm{kg}/m^{2}$)	-	(μg/ml)		
36	F	40	159	63	24.9	44	12.3	3.3	80.7
37	F	30	160	53	20.7	40	5.4	5.7	94.9
38	F	18	163	64	24.1	46	7.9	4.9	97.6
39	F	64	158	58	23.2	42	7.1	5.4	81
40	M	63	171	104	35.6	67	9.2	3.3	95.7
41	F	30	156	44	18.1	35	7.7	4.2	97.7
42	F	19	168	54	19.1	42	6.6	2.8	88.2
43	F	51	168	58	20.5	44	5.4	10	92.6
44	F	59	171	75	25.6	52	9.3	4.7	97.7
45	M	73	174	67	22.1	55	7	3.8	91.7
46	M	23	173	66	22.1	54	5.9	5.1	96.4
47	M	25	181	74.5	22.7	60	7.7	4.4	90.1
48	M	31	170	85	29.4	62	7.4	10	90.6
49	M	32	186	100	28.9	73	7.8	6	91.7
50	F	40	157	85	34.5	48	4.6	2.5	97.1
51	F	27	157	58	23.5	42	7.1	4	97.5
52	F	71	174	71	23.5	51	7.7	7.2	95.6
53	M	32	180	78	24.1	62	5.4	9.2	92.6
54	F	21	166	63	22.9	46	6.3	4.8	97.6
55	X	48	170	84	29.1	54	5.2	3.3	94.8
56	M	63	168	82	29.1	60	7.2	4.4	97.4
57	M	71	175	66	21.6	54	5.7	10	80.4
58	F	48	165	76	27.9	50	5.5	7.9	95.8
59	F	71	167	83	29.8	52	6.1	3.7	94.4
60	X	21	160	60	23.4	43	7.2	4.5	97.3
61	X	21	171	73	25	51	8.5	10	92.9
62	X	34	172	90	30.4	56	8.4	6.1	95.7
63	X	26	170	50	17.3	41	5.8	7.1	97.5
64	F	33	172	94	31.8	56	6.5	2.6	97.6
65	X	22	172	69	23.3	50	7.4	6.4	97.5
66	F	70	165	62	22.8	45	5.8	6.3	89.2
67	X	21	168	56	19.8	43	6	2.8	96.7
68	F	35	160	82	32	49	7.4	4.2	96.5
69	X	21	165	70	25.7	48	4.9	6.1	95.3
70	X	20	155	49	20.4	38	6.3	4	97.5

**Table C.11:** Coefficient interval bounds for female and male patients.

**Gender**	**Bound**	$a_{4}$	$a_{3}$	$a_{2}$	$a_{1}$	$a_{0}$
Female	$a_{i}^{-}$	1	0.7944	0.0802	0.0005	$2.5150\times 10^{-7}$
	$a_{i}^{+}$	1	1.8607	0.1481	0.0043	$2.504\times 10^{-6}$
						
Male	$a_{i}^{-}$	1	0.7480	0.0680	0.0004	$1.9391\times 10^{-7}$
	$a_{i}^{+}$	1	1.6074	0.1291	0.0035	$1.9669\times 10^{-6}$

**Table C.12:** Routh-Hurwitz stability check for all edge polynomials. All first-column entries are strictly positive, confirming internal stability.

**Gender**	**Polynomial**	$a_{4}$	$a_{3}$	$b_{1}$	$c_{1}$	$d_{1}$	**Stability**
Female	$p_{1}$	+	+	+	+	+	✓Hurwitz
	$p_{2}$	+	+	+	+	+	✓Hurwitz
	$p_{3}$	+	+	+	+	+	✓Hurwitz
	$p_{4}$	+	+	+	+	+	✓Hurwitz
							
Male	$p_{1}$	+	+	+	+	+	✓Hurwitz
	$p_{2}$	+	+	+	+	+	✓Hurwitz
	$p_{3}$	+	+	+	+	+	✓Hurwitz
	$p_{4}$	+	+	+	+	+	✓Hurwitz

## Risk relation approximation

Approximating the nonlinear relationship from Fig. 5 involves finding the parameters of a sum of sinusoidal functions that minimize the sum of the squares of the differences (residuals) between the data points and the curve defined by the proposed functions. At each iteration, the trust region algorithm approximates the objective function within a region around the current parameter estimate θ using a quadratic model:

(A.1) $$Q_{k}(p)=\overset{n}{\underset{i=1}{\sum }}{\left(r_{i}(\theta_{k})+\nabla r_{i}{\left(\theta_{k}\right)}^{⊤}p+\frac{1}{2}p^{⊤}\nabla^{2}r_{i}(\theta_{k})p\right)}^{2}\text{.}$$

The best approximation is determined using performance measurements. The results are shown in Fig. A.34 and Table A.5 where 70% of data is taken for identification and 30% for validation.

In Table A.5, SSE (Sum of Squares due to Error) measures the discrepancy between the data and an estimation model; a lower SSE indicates a better fit. R-square, also known as the coefficient of determination, indicates the percentage of the variance in the dependent variable (PP ratio) that is explained by the independent variables in a regression model; a value of 1 means a perfect fit. Root Mean Squared Error (RMSE) is the square root of the average of the squares of the errors. It represents the sample standard deviation of the differences between predicted values and the data. In order to choose the global best-fitting solution, the Normalized Root Mean Square Error (NRMSE) is calculated, having at least the NRMSE<0.1. A minimal error value close to 0 indicates a perfect fit between the data and the estimated model outputs, giving a fitting percentage equal to fit=100*(1-NRMSE).

The equation denoting the risk is given by:

(A.2) $$\overset{m}{\underset{j=1}{\sum }}a_{j}\sin(b_{j}\cdot \mathrm{BMI}+c_{j}),$$

and the identification results are summarized in Table A.6.

## Patients databases

For the 24 patient database, the clinical related information can be found on ClinicalTrials.gov/NCT00735631, and was compliant with the regulatory framework stated in the European Regulation (EU) 2017/745, and approved by the Ethics Committee of Ghent University Hospital, Belgium.

For the database of 70 patients, the clinical investigation involving human subjects was compliant with the regulatory framework stated in the European Regulation (EU) 2017/745. This academic clinical investigation was approved by the Ethics Committee of Ghent University Hospital and the Federal Agency for Medicines and Health Products of Belgium FAGG (EC/BC-08020, FAGG/80M0840, EudraCT: CIV-BE-20–07-0342442020, clinicaltrials.gov: NCT04986163).

## Stability using Kharitonov theorem

Kharitonov’s theorem [81] is a fundamental result in robust control theory, particularly useful for analyzing the stability of systems with parametric uncertainty. It is specifically designed for interval polynomials, where each coefficient can vary independently within a given interval. It offers a simple and powerful stability criterion.

Consider the family of polynomials:

$$P=\left\{p(s)=a_{0}+a_{1}s+a_{2}s^{2}+\ldots +a_{n}s^{n}\;|\;a_{i}\in [a_{i}^{-},a_{i}^{+}],\;i=0,1,\ldots ,n\right\}\text{.}$$

Kharitonov’s theorem states that: All polynomials in the family P are Hurwitz stable *if and only if* four specific polynomials (the Kharitonov polynomials) are Hurwitz stable.

This result enables robust stability analysis across the entire parameter space without the need for exhaustive testing of all possible coefficient combinations. The stability of 16 is then confirmed using this theorem.

For each of the admissible patients presented in Fig. 7 and Eq. 18, we evaluated the coefficients $a_{4},\ldots ,a_{0}$ in 17, and extracted the minimal and maximal values to construct interval bounds $\left[a_{i}^{-},a_{i}^{+}\right]$. The process was repeated separately for each gender (set), as LBM is computed differently. By constructing coefficient bounds across the clinically admissible patient domain, we defined an interval polynomial family that accounts for inter-individual variability in drug kinetics. Applying Kharitonov’s theorem to this family provides a means to assess whether the internal stability of the PK model is preserved across the entire population, without the need for exhaustive stability checks for each patient. After the calculation, the results are shown in Table C.11.

Given interval bounds for each coefficient $a_{i}\in [a_{i}^{-},a_{i}^{+}]$, four Kharitonov polynomials (for each set) are defined as follows:

(C.1) $$\begin{array}{cc} p_{1}(s) & =a_{0}^{-}+a_{1}^{-}s+a_{2}^{+}s^{2}+a_{3}^{+}s^{3}+a_{4}^{-}s^{4},\\ p_{2}(s) & =a_{0}^{+}+a_{1}^{+}s+a_{2}^{-}s^{2}+a_{3}^{-}s^{3}+a_{4}^{+}s^{4},\\ p_{3}(s) & =a_{0}^{+}+a_{1}^{-}s+a_{2}^{-}s^{2}+a_{3}^{+}s^{3}+a_{4}^{+}s^{4},\\ p_{4}(s) & =a_{0}^{-}+a_{1}^{+}s+a_{2}^{+}s^{2}+a_{3}^{-}s^{3}+a_{4}^{-}s^{4}\text{.} \end{array}$$

These polynomials represent the vertices of the coefficient space defined by the intervals and are sufficient to assess robust Hurwitz stability for the entire family.

To rigorously verify the internal stability of each Kharitonov polynomial, we applied the Routh-Hurwitz stability criterion [82]. This classical method determines whether all roots of a polynomial lie in the open left-half of the complex plane by inspecting the signs of the first column of its Routh array, without computing the roots explicitly. The system is deemed asymptotic stable if and only if all elements in the first column are nonzero and exhibit no sign changes, thereby ensuring that no poles reside in the right-half plane.

For a fourth-order polynomial in Eq. (16), the Routh-Hurwitz array is constructed as follows:

$$\begin{array}{ccc} s^{4}a_{4} & a_{2} & a_{0}\\ s^{3}a_{3} & a_{1} & 0\\ s^{2}b_{1} & b_{2} & \\ s^{1}c_{1} & 0 & \\ s^{0}d_{1} & & \end{array}$$

with:

$$\begin{array}{cc} b_{1} & =\frac{a_{3}a_{2}-a_{4}a_{1}}{a_{3}},\;b_{2}=\frac{a_{3}a_{0}}{a_{3}},\\ c_{1} & =\frac{b_{1}a_{1}-a_{3}b_{2}}{b_{1}},\;d_{1}=\frac{c_{1}b_{2}}{c_{1}}\text{.} \end{array}$$

We applied this criterion to each of the four Kharitonov polynomials per gender, resulting in the following stability verification table in C.12.

For both genders, all four polynomials were verified to be Hurwitz. The entire family of fourth-order patient-specific PK models is then guaranteed to be internally stable across the full range of clinically admissible patients, regardless of gender, age, height, weight, or BMI.
