# Supplementary material for: Pharmacokinetic modelling during long-term anesthesia: minimizing the gap
Source: J Adv Res. 2025 Jun 24;82:535–61. doi: 10.1016/j.jare.2025.06.047 (PMC13001178; doi:10.1016/j.jare.2025.06.047)
Supplement: Supplementary file 1 [file mmc1.pdf]

Pharmacokinetic Modelling During Long Term Anesthesia: Minimizing the Gap

Amani R. Ynineb, Erhan Yumuk, Dana Copot, Ghada Ben Othman, Hamed Farbakhsh, Isabela Birs, Robin De Keyser, Samir Ladaci, Cristina Muresan, Martine Neckebroek, Clara M. Ionescu

Study cases investigated in this paper for the closed loop control.

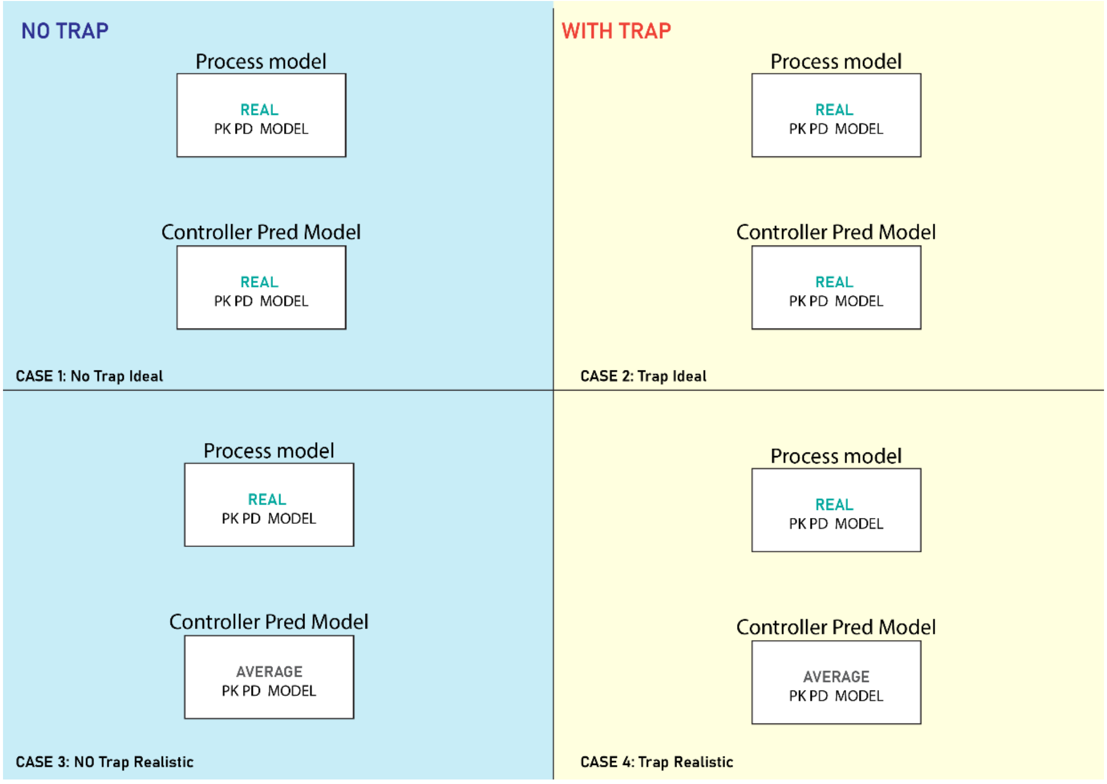

Figure 1: Visual representation of the 4 cases

Statistical analysis for all study cases.

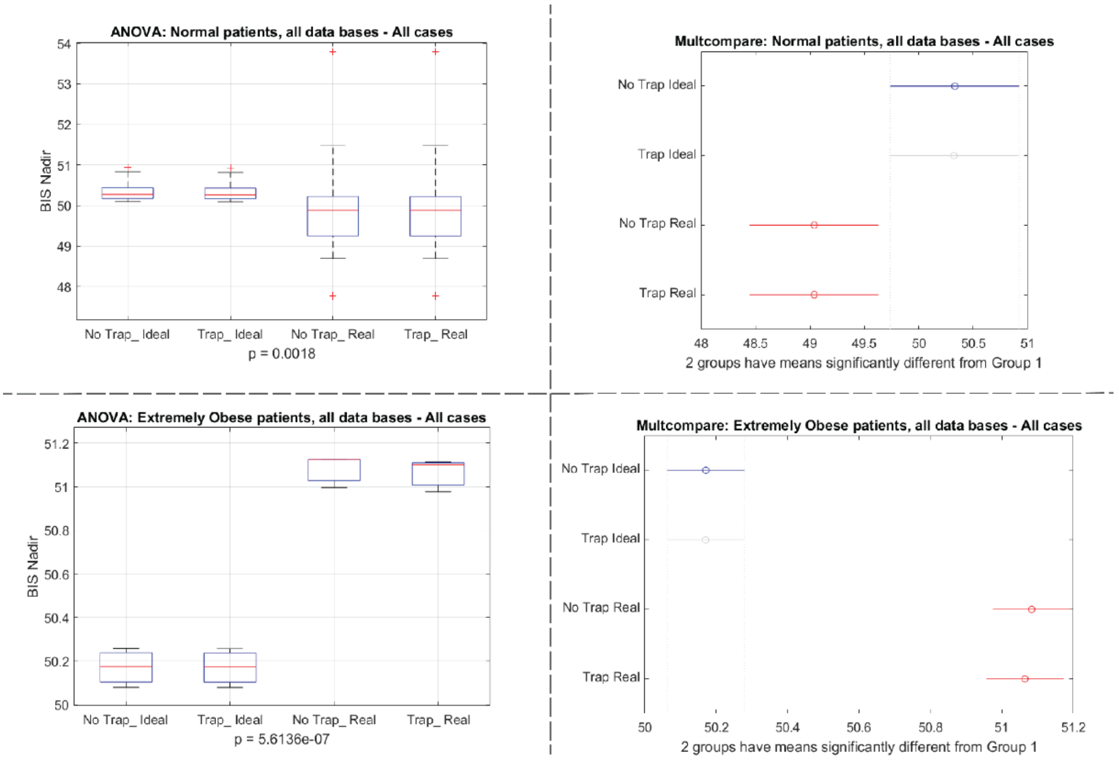

Figure 2: ANOVA Analysis (left) and Multcompare test (Right) of BIS Nadir in Normal BMI (Top) and Extremely Obese (Bottom) for all cases

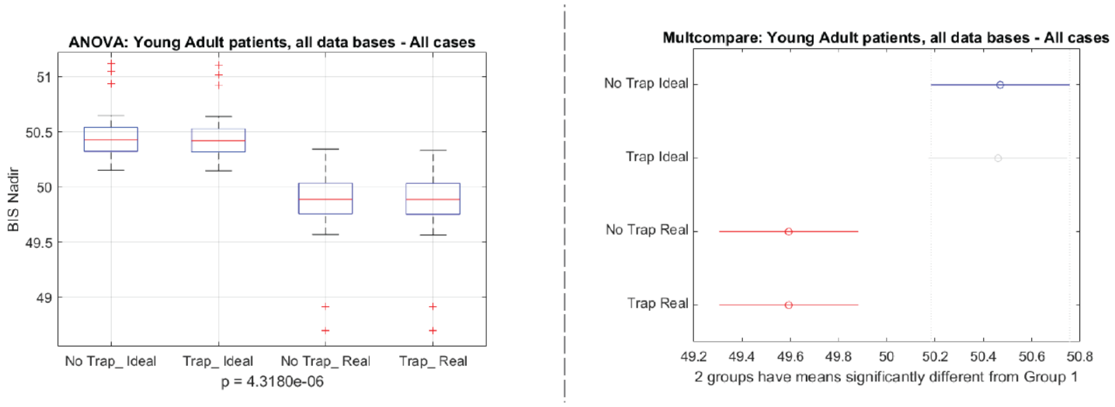

Figure 3: ANOVA Analysis (left) and Multcompare test (Right) of BIS Nadir in young adult patients for all cases

MATLAB PROGRAM FOR OPEN-LOOP ANALYSIS

```
close all
clear all
clear memory

% Openloop simulation of a drug bolus

% ---------------------------------------------------------------%
%%%%%%%%%%%%%%%%%%%%%%%%  initialisation  %%%%%%%%%%%%%%%%%%%%%%%%
% ---------------------------------------------------------------%
h=1/60;               % sampling time 1 second (in minutes)
Tsim=72;               % Total simulation time  (in hours)
t=0:h:Tsim*360*h;     % Time vector
n=round(t/h);         % simulation samples
dose=3.33*5;          % impulse of injection 3,33 mg/s during 5 sec

Comp1_all=[]; Comp2_all=[]; Comp3_all=[]; CompE_all=[]; CompT_all=[];
BIS_all=[]; %% saving vectors

% ---------------------------------------------------------------%
%%%%%%%%%%%%%%%%%%%%%% 12 patient database  %%%%%%%%%%%%%%%%%%%%%%
% ---------------------------------------------------------------%
         % index age height weight gender BMI   lbm C50   gamma
 Patients12=[
             1    40  163     54      2     20.3  42  6.33  2.24 ;
             2    36  163     50      2     18.8  40  6.76  4.29 ;
             3    28  164     52      2     19.3  41  8.44  4.1  ;
             4    50  163     83      2     31.2  50  6.44  2.18 ;
             5    28  164     60      1     22.3  49  4.93  2.46 ;
             6    43  163     59      2     22.2  44  12.1  2.42 ;
             7    37  187     75      1     21.4  62  8.02  2.10 ;
             8    38  174     80      2     26.4  54  6.56  4.12 ;
             9    41  170     70      2     24.2  50  6.15  6.89 ;
             10   37  167     58      2     20.8  44  13.7  1.65 ;
             11   42  179     78      1     24.3  61  4.82  1.85 ;
             12   34  172     58      2     19.6  45  4.95  1.84];

[m,n]=size(Patients12);

% ---------------------------------------------------------------%
%%%%%%%%%%%%%%%%%%%%%% Loop For each Patient  %%%%%%%%%%%%%%%%%%%%%%
% ---------------------------------------------------------------%
for k=1:m
    age =Patients12(k,2); height =Patients12(k,3); weight =Patients12(k,4);
Gender =Patients12(k,5); BMI =Patients12(k,6);
    lbm =Patients12(k,7); c50=Patients12(k,8); gama=Patients12(k,9);


    %%%%% PK Model %%%%%
    clear1=1.89+0.0456*(weight-77)-0.0681*(lbm-59)+0.0264*(height-77); %
clearance for the blood
    clear2=1.29-0.024*(age-53)       % clearance for the muscle
    clear3=0.8                       % clearance for the fat
```

```
    %%%%%  volumes of distribution for each compartment %%%%%
    V1=4.27;                     % central compartment; [L]
    V2=18.9-0.391*(age-53);      % muscle compartment [L]
    V3=238;                      % fat compartment [L]

    k21m=clear2/V2; k12m=clear2/V1; k31m=clear3/V3; k13m=clear3/V1;    % 1/min
    k10m= clear1/V1;                                                   % 1/min


    %%% Trap fat model %%%%

    traprisk = 27.4472*sin(0.0033*BMI-0.0570)+0.8002*sin(0.5390*BMI-4.2180)+...
               0.4815*sin(0.7595*BMI-6.2212);

    Vt=BMI*V3/100;                     %%% percent as a fcn of BMI
    clearX=0.852/traprisk;             %%% clearance as relative BMI to lbm ratio
    k3tm=clearX/Vt; kt3m=clearX/V3;    %%% TRAP Coefficient %%%%


    %%% effct site compartement
    E0=100;
    Emax=100;
    ke0=0.459;
    k1e=ke0;

    q1=dose;                        % where the bolus injection is given in blood
    q2=0;    q3=0;    qt=0;    qe=0; % this is 0 because there is no drug yet

    cp1=1; cp2=1; cp3=1; cpe=1; cpt=1;
    Comp1=[];Comp2=[]; Comp3=[]; bis=[]; CompE=[]; CompT=[]; Y=[];
    Comp1n=[];Comp2n=[]; Comp3n=[]; bisn=[]; CompEn=[];

    for j=1:1:length(t)
         c1(j)=(1-2/j)*cp1; %%%% Blood
         cp1=c1(j);
         c2(j)=(1-2/j)*cp2; %%%% Muscle
         cp2=c2(j);
         c3(j)=(1-2/j)*cp3; %%%% Fat
         cp3=c3(j);
         ce(j)=(1-2/j)*cpe; %%%% Effect site
         cpe=ce(j);
         ct(j)=(1-2/j)*cpt; %%%% Fat Trap
         cpt=ct(j);
   end

       %%%%%  these are the equations of the PK model plus gain Hill curve %%%%%
   for i=2:1:length(t)
        q1(i) =(k21m*q2(i-1)+k31m*q3(i-1)-k12m*q1(i-1)-k13m*q1(i-1)-k10m*q1(i-
1))*h-memo(q1,c1,i); %%%% Blood
        q2(i) =(k12m*q1(i-1)-k21m*q2(i-1))*h-memo(q2,c2,i);
%%%% Muscle
        q3(i) =(k13m*q1(i-1)-k31m*q3(i-1)-k3tm*q3(i-1)+kt3m*qt(i-1))*h-
memo(q3,c3,i);              %%%% Fat
        qt(i) =(k3tm*q3(i-1)-kt3m*qt(i-1))*h-memo(qt,ct,i);
%%%% Fat Trap
        qe(i) =(k1e*q1(i)-ke0*qe(i-1))*h-memo(qe,ce,i);
%%%% Effect site compartment
        out(i)=E0-Emax*(qe(i)^gama)/(c50^gama+qe(i)^gama);      %%%% BIS
   end
```

```
     for j=1:1:length(t)
        Y(j,1)= q1(j) ; %%%% Blood
        Y(j,2)= q2(j) ; %%%% Muscle
        Y(j,3)= q3(j) ; %%%% Fat
        Y(j,4)= qe(j) ; %%%% Effect site compartment
        Y(j,5)= out(j); %%%% BIS
        Y(j,6)= qt(j) ; %%%% Fat Trap
    end

    T=[0:1:length(t)-1]; % in seconds
    Comp1=[Comp1 Y(:,1)];Comp2=[Comp2 Y(:,2)]; Comp3=[Comp3 Y(:,3)];
    CompE=[CompE Y(:,4)]; bis=[bis Y(:,5)];
    CompT=[CompT Y(:,6)];

    %%%%%  save variables %%%%%
    Comp1_all=[Comp1_all, Comp1]; Comp2_all=[Comp2_all, Comp2];
    Comp3_all=[Comp3_all,Comp3];
    CompE_all=[CompE_all,CompE]; CompT_all=[CompT_all,CompT];
    BIS_all=[BIS_all,bis];

end %%% Patients loop

% --------------------------------------------------------------%
%%%%%%%%%%%%%%%%%%%%%%%%%%%% Plots  %%%%%%%%%%%%%%%%%%%%%%%%%%%%%
% --------------------------------------------------------------%

figure;
grid on

subplot(321); plot(T./360,Comp1_all, 'Color', [0.64, 0.08, 0.18]);ylabel('x_1
(mg/L)')
grid on; hold on

subplot(323); plot(T./360,Comp2_all,'k');ylabel('x_2 (mg/L)')
grid on; hold on

subplot(325); plot(T./360,Comp3_all,'Color',[0.4660, 0.6740, 0.1880])
;ylabel('x_3 (mg/L)')
grid on; hold on

subplot(322); plot(T./360,CompE_all, 'Color', [0.4940, 0.1840,
0.5560]);ylabel('x_e (mg/L)')
grid on; hold on

subplot(326); plot(T./360,CompT_all,'Color',[0, 0.75, 0.75]); ylabel('x_t
(mg/L)')
grid on; hold on

subplot(324); plot(T./360,BIS_all,'b');ylabel('BIS (%)')
grid on
%%%%%%%%%%%%%%%%%%%%%%%%%%%%%%%%%%%%%%%%%%%%%%%%%%%%%%%%%%%%%%%%%%%
```

Memo function

```
function [y]=memo(r,c,k)
temp=0;
for j=1:k-1
    temp=temp+c(j)*r(k-j);
end
y=temp;
```
